# Care preferences of older migrants and minority ethnic groups with various care needs: A scoping review

**DOI:** 10.1371/journal.pone.0341147

**Published:** 2026-01-23

**Authors:** Viktoria Peters-Nehrenheim, Mike Rommerskirch-Manietta, Hürrem Tezcan-Güntekin, Martina Roes

**Affiliations:** 1 German Center of Neurodegenerative Diseases (DZNE), Site Witten, Germany; 2 Faculty of Health, Department of Nursing Science, University of Witten/Herdecke, Witten, Germany; 3 Alice Salomon University of Applied Sciences Berlin, Berlin, Germany; Paris School of Business, FRANCE

## Abstract

**Background:**

Older migrants and people from minority ethnic groups face unique challenges in accessing healthcare. Various factors often lead to lower healthcare utilization and hinder person-centered care. However, little is known about the various care preferences of these people, and no systematic overview of their care preferences has been conducted.

**Methods:**

We conducted a scoping review in accordance with the description provided by the Joanna Briggs Institute. We searched the MEDLINE, CINAHL, PsycINFO, and Cochrane Library databases and extracted data from the included publications. We subsequently identified and analyzed the care preferences of older migrants and minority ethnic groups (people aged 60 years or older with various care needs) via inductive content analysis in an iterative process.

**Results:**

Initially, 2756 records were identified through our electronic database search. After duplicate removal, 1924 records were screened for relevance. A total of 173 reports remained for full-text screening. A total of 50 studies published from 1985–2024 were included in the review. Our analysis of these articles revealed descriptive themes that were grouped into four main categories: I) care practice; II) professionals; III) living with others; and IV) environment. These main categories were further divided into 16 subcategories that captured the identified preferences.

**Conclusion:**

Our research yielded two key findings. First, preferences related to care are as heterogeneous as the people receiving care. Second, people with dementia and a history of migration and their preferences in the nursing context, have been underrepresented in healthcare research. To bridge this knowledge gap, future research should prioritize understudied populations. By examining these groups, we can gain a more comprehensive understanding of the multifaceted individual experiences of older migrants and people from minority ethnic groups.

## Introduction

It is estimated that there were approximately 281 million international migrants worldwide in 2020, which equates to 3.6% of the global population [1]. Globally, 33.7 million people with a migration background were 65 years or older in 2020 [2]. Older people with a migration background face different challenges in accessing health care, and caring for these individuals is a major worldwide public health challenge. In the future, the number of older people in need of care will increase, including those with a migration background [3].

Interestingly, there is no common definition of the term “migrant”, which has varying definitions and is often used as an umbrella term for people who leave (temporarily or for the long term) the place where they were born [4–8]. In contrast, “immigrant”, which is not a term commonly used in all countries, refers to all people who move from their home country to another country willingly and legally and apply for permission to enter and live in the country permanently, which allows them to work in the new country without any restrictions (permanent residency) [5]. Migration has contributed to increasing the richness of diverse cultures and ethnicities worldwide. However, migrants and immigrants are not the only sources of diversity in society [9,10]. The term ‘minority ethnic group’ usually refers to a group of people who share a common cultural identity that differs from that of the local population (in a particular country) [4,10,11]. An examination of the various examples and definitions reveals that being part of a minority ethnic group does not necessarily mean that one is an immigrant; however, the use of words in different contexts or specifications of categories within different research studies may blur this distinction. In practice, however, the existing terms have different meanings. Minority ethnic groups or cultural minorities may include migrants who become part of the established minority ethnic groups in the countries to which they migrate. Alzheimer Europe defines ethnicity as “a shared culture, often incorporating a common language, geographic locale or place of origin, religion, sense of history, traditions, values, beliefs and food habits”. The term ethnic group refers to a group of people who have common cultural traits. The traits of these groups are distinct from those of other groups. People who share a common language, place of origin, religion, traditions, values, beliefs, food habits, and so forth are perceived and view themselves as an ethnic group because they share the same ethnic principles [24]. At this point, however, it should be noted that not all migrants belong to minority ethnic groups and that not all people from minority ethnic groups are migrants [12].

In our review, we use the term “migration” as an umbrella term that refers to human mobility across national borders (international migration), and the term involves the process of both coming to one country and leaving another country. Migration is more than the actual time at which an individual enters into the national territory of a country that differs from the country in which he or she is a member because of his or her birth within the national borders. Migration is a process that begins with arriving into a country but continues as long as individuals remain in a foreign country and hold an alien or foreign resident status. In this context, “the endurance of a migrant status for those who were born on national soil (so-called second- and third-generation migrants) and the effect of naturalization raise the issue of whether and how holding papers and citizenship rights alter one’s self-perception and social categorization and thus affect identity formation” [13].

Migrants face many barriers to accessing health care that are linked to their distinct lifestyles, language challenges, particular attitudes, and cultural differences or values [6,14–16] when they migrate from one country to another, often leading to unmet needs and poor quality of care [17,18]. Although people from minority ethnic groups experience similar levels of stress, minority ethnic groups are often recognized but not necessarily accepted by the larger society in which they reside, which may lead to disadvantages in everyday life situations [18,19]. People with a migration background as well as people from minority ethnic groups have different healthcare needs, different care traditions, and different understandings of illness and treatment than the local population does [19,20]. Individual migrants and people from minority ethnic groups bring their values, beliefs, and preferences from their home culture to their new country of residence, and these values, beliefs, and preferences are integral to the process of relating to new social contexts [21]. This construct of acculturation can be defined as the “process of learning and incorporating the values, beliefs, language, customs and mannerisms of the new country”. This also includes behaviors that affect health and activity levels [22].

In 2000, Carpenter et al. reported that older adults with functional limitations need assistance ranging from traditional activities of daily living to more abstract expressions of their personality. This means that people with migration a background and people from minority ethnic groups have unique ideas regarding care needs and different thoughts and feelings regarding how care needs should be met [23]. Knowledge of the individual everyday life preferences of older people in need of care is the foundation of person-centered care [24,25], which leads to positive care outcomes. Person-centered care can be defined as “providing care that is respectful of and responsive” to individual preferences, needs, and values, and it has a positive influence on a person’s well-being and satisfaction with care [26,27]. Therefore, knowledge of the individual as a whole person is necessary, involving them—and when appropriate, their family and friends—in helping to assess their own needs and plan their own care [28].

The concept of preferences can be described as “an expression of the attractiveness of an option that serves to fulfill a person’s *needs*, is determined on the basis of one’s *values* and directs behaviors to achieve *goals*” and is an “integral measurement tool to operationalize the more abstract constructs (i.e., needs, values, goals) of individuals” [24]. Van Haitsma et al. reported that preferences cover a broad range of behaviors and activities related to daily life, such as “leisure activities, caregivers and care, social engagement, and activities of daily living” [24]. To date, little is known about the care preferences of older migrants and people from different minority ethnic groups, and no systematic overview of the various care preferences of older migrants and people from minority ethnic groups has been conducted. A lack of familiarity with the health care system, different perceptions, and cultural and language barriers often result in lower use of health care services, and unknown care preferences hinder person-centered care [29]. In addition, a systematic overview would allow us to identify potential research gaps and/or to further develop the concept of preferences. Our scoping review aims to fill this gap and describe the current research landscape for a specific topic in a user-friendly and visual way.

## Research question

In the context of a pilot study focusing on the translation and psychometric testing of an instrument for assessing the preferences for everyday living of older people in various care settings [30–33], the results clearly indicate that migrants and people from minority ethnic groups have not been considered from the perspective of older adult care recipients.

In this context, the authors of this review discussed questions regarding the population of diverse groups. Our discussion focused especially on older migrants and people from minority ethnic groups and what care preferences they may have. Then, we carried out an explorative search to provide a systematic overview of the currently described care preferences of older migrants and people from minority ethnic groups. To date, no systematic overview has been provided. On the basis of these results, we defined the following research question for our scoping review: “What are the care preferences of older migrants and people from minority ethnic groups that can be identified in the literature?”

## Methods

**Fig 1:**
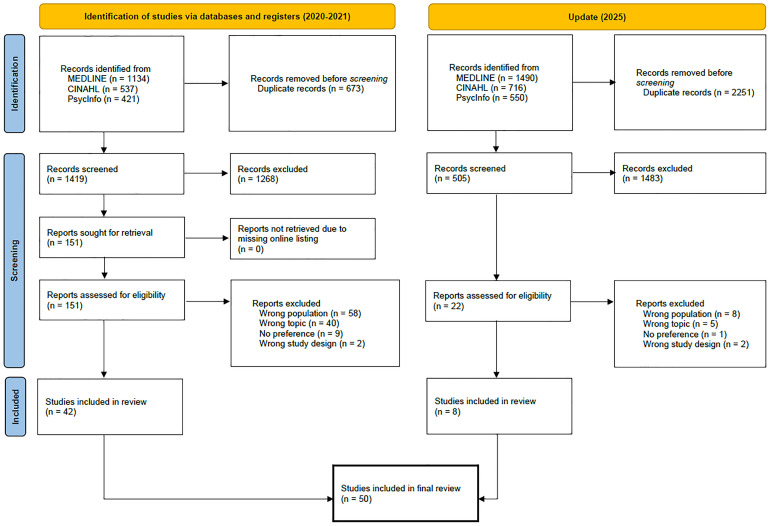
PRISMA 2020 flow diagram [36] demonstrating the identification, screening, and eligibility of studies for inclusion in the scoping review.

For our scoping review, we published a study protocol describing our methodological approach [34]. We used the Preferred Reporting Items for Systematic reviews and MetaAnalyses extension for Scoping Reviews Checklist [35] and the flow chart of the updated Preferred Reporting Items for Systematic Reviews and Meta-Analyses guidelines [36] to conduct our scoping review (S5 Table and Fig 1). This scoping review is based exclusively on the analysis of published peer-reviewed and grey literature. As no primary data were collected and no human participants were directly involved, ethical approval was not required.

### Literature search

We chose the scoping method for this review because we wanted to provide a broad overview of the different types of preferences related to older migrants and people from minority ethnic groups with various care needs, as research in this field is limited. Therefore, the first author (VPN) conducted a preliminary limited search in MEDLINE (via PubMed) to obtain a broad understanding of the different understandings and uses of the term “preferences” and to generate an initial understanding. We identified other key terms in addition to “preferences”, as the papers used analogies to describe the topic, e.g., needs, beliefs, values, expectations and wishes. The different topics were operationalized by three researchers (VPN/MRM/MR) into a combination of index search terms and keywords. Our search string was developed by one researcher with a migration background (VPN) and was checked by the whole research group (VPN/MRM/DP/MR/HTG) according to the Peer Review of Electronic Search Strategies (PRESS) recommendations [37]. The search string was developed first for MEDLINE (via PubMed) and was modified by one researcher (VPN) for the Cumulated Index to Nursing and Allied Health Literature (CINAHL) (via EBSCO) and PsycINFO (via EBSCO) according to the description in RefHunter Vers. 5.0 [38]. The search strategies for MEDLINE (via PubMed), PsycInfo (via EBSCO), and CINAHL (via EBSCO) are provided in S2, S3 and S4 Tables. Between November 2021 and February 2025, we searched the following electronic databases: MEDLINE (via PubMed), CINAHL (via EBSCO) and PsycInfo (via EBSCO).

### Study selection

In the first step, the first author (VPN) imported the records identified in our electronic database search into Covidence [39] and automatically checked for duplicates. In the second step, all titles and abstracts of the identified records were screened in Covidence by three researchers (VPN/MRM/DP) against the inclusion and exclusion criteria (Table 1). Discrepancies in voting were discussed and resolved in regular (video) meetings. Third, full-text screening was conducted by the same three researchers (VPN/MRM/DP); differences were discussed and resolved in regular (video) meetings.

**Table 1:** Inclusion and exclusion criteria.

Criteria	Definition
***Population***	We considered studies with a study population of older migrants and people from minority ethnic groups (mean or median age ≥ 60 years [40] with various care needs (with and without a dementia diagnosis)) across care settings. Various care needs were defined as a need (specified or unspecified) for daily nursing care regardless of the setting in which a person lives or resides (e.g., community, home care, nursing facility, or hospital). Migrants were defined based on their country of birth (people with a migration background are those living in countries other than their country of birth) [7]. We did not exclude studies in which professionals or family caregivers reported the preferences of older individuals (proxy-reported). We excluded studies that focused on people receiving palliative care and/or with a focus on the end-of-life preferences of older migrants and people from minority ethnic groups [41].
***Concept of interest***	We considered studies that described the various care preferences of older migrants and people from minority ethnic groups. In this context, terms related to the term “preferences” were also considered relevant. We conducted a broad search, which included other common terms and synonyms for preferences that we found in an initial limited search. This approach increased the probability of finding the most relevant literature, as well as the literature that did not use the term “preferences” in the title or abstract to define the research findings. In the full-text screening, not only was the term “preferences” considered relevant for inclusion or exclusion, but the following terms were also considered relevant: preferences care expectations care wishes care needs values demands
***Study design***	Because the objective was to identify the existing care preferences of older migrants and people from minority ethnic groups in the literature, the searches focused on peer-reviewed scientific empirical research papers. We included all study types. We excluded discussion papers or mission statements, conference abstracts, and editorials.
***Others***	There were no restrictions on publication status or date. We excluded all studies that were not published in English or German.

### Data charting process

Our data extraction form was based on the template for scoping reviews developed by the Joanna Briggs Institute [42], was created in Microsoft Word and included the following columns: study characteristics such as the study type (study design/methodology) and setting (healthcare context, country of origin, and study period); population characteristics (sample size, age, and sex); and study aim and preference characteristics. Data extraction was performed by one researcher (VPN) and randomly checked for consistency by another researcher (MRM) after extraction was completed. If deviations between the extracted data and the reported data in the studies were identified, they were discussed and resolved in regular (virtual) meetings between the two researchers (VPN/MRM). The data extraction results were discussed by three researchers in a meeting (VPN/MRM/MR) with a focus on implications for subsequent data synthesis.

### Analysis

A qualitative content analysis via an inductive approach was performed to identify the main preferences reported in the included studies [43–45]. A combination of in vivo (i.e., verbatim) [46] and descriptive coding [47] (i.e., summarizing the meaning of the extracted text into a word or short phrase) was used to analyze the included studies [48].

To ensure transparency and methodological thoroughness, the coding and analysis process was conducted in several steps. First, one researcher (VPN) developed an initial draft code tree based on three to four studies, while a second researcher (MRM) independently created another draft code tree based on three to four studies. Both researchers then compared and discussed their preliminary code trees to identify overlaps and differences. After iterative discussions, consensus was reached on a final coding structure, which served as the foundation for the subsequent content analysis. The main analysis was then carried out by VPN using the agreed-upon code tree, and additional relevant preferences and categories that emerged during the iterative coding process were identified. MRM randomly checked the updated coding results for consistency and accuracy. In cases where the categorization was unclear, the relevant codes were discussed among the research team (VPN/MRM/MR) until agreement was achieved.

### Mapping of preferences

We used the extracted data to synthesize and map the identified care preferences.

Using Microsoft Word, we created a table (S1 Table) that provides descriptions of the included studies in a narrative style to map the various characteristics of the identified preferences. S1 Table synthesizes the data into a summarized overview (e.g., primary publication, study characteristics, population, and setting) and provides general information on the identified types of preferences.

## Results

We initially identified 2756 records through our electronic database search. After removing duplicates, 1924 records were screened for relevance (title and abstract; using the inclusion and exclusion criteria). A total of 173 reports were retained for full-text screening. A total of 50 studies were included in the review, and the characteristics of the included studies are shown in S1 Table [49–99]. Fig 1 illustrates the identification, screening, and eligibility assessment of studies prior to their inclusion in our scoping review, including the reasons for exclusion.

### Study characteristics

This review includes studies involving a range of methodologies: 32 studies with a qualitative design, 9 studies with a quantitative design, five reviews, and three studies with a mixed method design. The number of studies included according to continent are as follows: North America (n = 30), Europe (n = 12), Australia (n = 8), and South Africa (n = 1). These studies were published during the period of 1985–2024. Table 2 shows the populations of older migrants and people from minority ethnic groups in the specific countries. In the included studies, different terms and descriptions of ethnicity and race were used. Furthermore, “the characteristics that define ethnicity are not standardized, so the use of ethnicity is imprecise and fluid” [100]. Common terminology for ethnic minority populations (Asian individuals, Black individuals, Chinese individuals, etc.) may suffice for everyday conversation or in a political context but is insufficient to conduct a structured search strategy [100]. We are aware that different labels refer to different populations depending on the terminology used in different countries. Therefore, in this review, we refer to populations as mentioned in the original studies. In S6 Table, we provide an overview to ensure conceptual clarity and consistency in terminology. This summary table outlines how key population terms (“migrants,” “immigrants,” and “minority ethnic groups”) were used in the included studies and enables readers to better understand how these terms were applied and defined across different contexts and study settings.

**Table 2:** Study populations.

(Immigrated to:) Country	Study population	
**USA**	**African American** [59,62,63,66,68,82,84,87] and Black Ethnic Minority [57] **American Indians** (natives) [53,55] **Arabic-speaking** immigrants [49] **Chinese American** [50,51,70,84,89,93] **Cuban immigrants** [79] **Hispanics** [58] **Latinos/Mexican-American**[62,68,74,82,84]	**Asian Indians** [86,93] **Japanese American** [64,67,93] **Korean Americans** [69,73,85,93] **Filipino** [93] **Pakistani** [93] **Vietnamese** [93] **Bangladeshi** [93] **Taiwanese** [93]
**CANADA**	**Chinese Canadians** [56,90] **Filipin**o [78]	**Japanese Canadians** [72] **Punjabi-speaking South Asian** (Indian, Pakistani) [71]
**AUSTRALIA**	**Chinese Australians**[54] **Germans** [80,99] **Italian** [99]	**Greeks** [65,99] **Persons from diverse cultures**[83,95] **Ethnic Minority** [98]
**UNITED KINGDOM**	**African Caribbean** [60,77] **Bangladeshi**[60,77,81] **Chinese Ethnic Minority** [77] **Hungarian** [60] **Indian** [60] **Chinese Minority** ethnic carers of older people in the UK n = 1 [77]	**Italian** [60] **Pakistani Immigrants** [60,77] **Polish** [60] **Ukrainian** [60] **Other Asian Minority** [77]
**NETHERLANDS**	**Moroccan** [88] **Surinamese** [88] **Turkish** [88]	
**SWEDEN**	**Sami** in Sweden (indigenous) n = 1 [76]	
**NORWAY**	**Pakistani** [52] **Sami** in Norway (indigenous) n = 1 [61,94]	
**NEW ZEALAND**	**Filipino** [75]	
**SOUTH AFRICA**	**Ethnic Norwegians**[61]	

**Fig 2:**
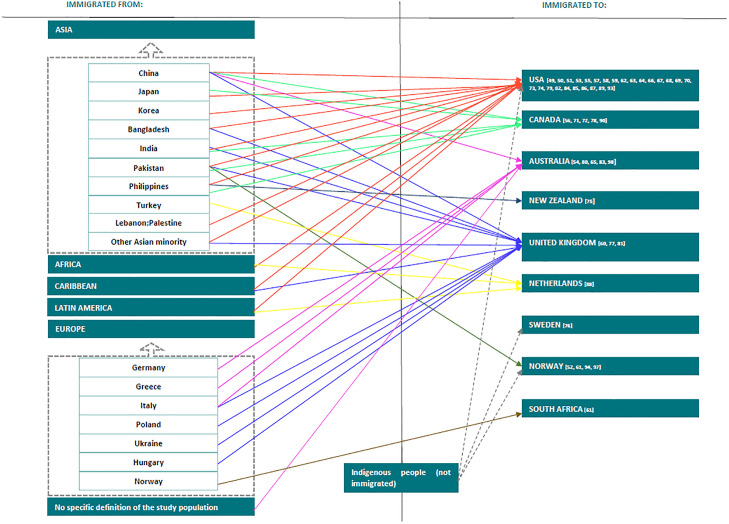
Study populations.

The 50 selected studies included diverse populations of older migrants with many different origins or ethnicities. The migratory destinations of the identified study populations are shown in Fig 2.

We identified extraordinarily strong migratory flows to the United States of America (USA), Canada, Australia, and the United Kingdom (UK). Older migrants migrated to the USA from China, Japan, Korea, Taiwan, Cuba, India, Lebanon/Palestine, Africa, the Philippines, the Caribbean, and Latin America. Older migrants from China, Bangladesh, India, Pakistan, Italy, Poland, Ukraine, Hungary and other, not specifically named, parts of Asia migrated to the UK. Additionally, a large migratory flow of older migrants to Canada was observed, where older migrants emigrated from China, Japan, India, Pakistan, and the Philippines. In our review, we found that older migrants from China, India, and Pakistan most frequently emigrated to various destination countries. Not all populations from the literature reviews could be included, as some reviews were very extensive, and their extraction would have exceeded the scope of this review. However, through the other included studies, a comprehensive overview of population migration flow was obtained; thus, we determined that including the information from the other studies would not have yielded significant changes.

### Identified care preferences

Our analysis of the 50 articles revealed descriptive themes that were grouped into four main categories: I) care practice; II) professionals; III) living with others; and IV) environment. The four main categories were further divided into 16 subcategories that capture the identified preferences (Table 3).

**Table 3:** Grouped categories referring to the major types of identified preferences.

Categories of preferences	Subcategories
**I. Care Practice**	Western vs. traditional medicine Care service models Housekeeping service Medication Activities Food
**II. Professionals**	Interpreter Sensitive to other cultures Biological sex Language Ethnicity
**III. Living with Others**	Living arrangements Informal care Ethnicity of the community
IV. Environment	Cultural familiarity Facility character

### Preferences within the care practice category

Nursing is defined by caring. Caring is a mutually beneficial experience for both patients and nurses as well as family caregivers. Caring can save the life of a patient and convey trust and commitment to patients, families, and staff. We defined our first main category, *care practice,* as support delivered by specially trained carers as well as family caregivers, different care service models, ethical inner values, and other diverse institutional or personal nursing practice standards.

#### Principles of medicine.

The subcategory *principles of medicine* focuses on Western vs. traditional medicine. For example, in Chinese medicine, diagnoses are performed on the basis of the symptoms the patient describes and the appearance of the patient (such as the eyes, skin, or tongue color as well as the pulse); then, the overall systemic problem is addressed, with a focus on preventing any potential adverse effects. In contrast, in Western medicine, the focus is primarily on symptoms, which are often treated in an isolated manner instead of considering the whole interconnected body system.

We identified a preference *for Western [over Chinese] medicine* [51,56,71] among older Asian migrants who speak the native language, as evidenced by observations within their cultural group. We identified a preference for traditional Chinese medicine [90], especially for acupuncture and herbal teas, but the use depends on financial resources because traditional medicine is not covered by health insurance.

#### Principles of care service models.

The subcategory *care service models* characterizes types of care models or types of care that are based on who provides the service and where the service is delivered. We identified diverse preferences with tendencies toward one’s own cultural background and toward the adopted cultures of the country of migration Table 4.

**Table 4:** Identified preferences for diverse care service models with different tendencies.

Tendency to country of origin	Tendency to country of migration
Preference for a culturally specific service (e.g., language specific, activity programs, food) [65,83] Preference for self-care over professional care (Chinese elders) [51] Preference for home respite service (carers from different minority ethnic groups) only when those they were caring for were well looked after in a culturally familiar setting (“preference for the persons they are caring for to be with people of the same ethnic origin and to receive the food they are used to”) [77] Preference for traditional Chinese care (influencing factors: religious beliefs and preference for traditional Chinese medicine; distinctive culture is related to the use of traditional care) [56] Preference for Japanese care (caring for elders as a calling and showing respect) [64] Preference (older Latino people) for family members to accompany elders to appointments with health care professionals (“someone outside the family would not fill the role of caregiver”) [82] Preference to maintain traditional practices and uphold Filipino spiritual and cultural beliefs (e.g., attending church, prayer rooms, Filipino staff, Filipino food) [75] Preference for a home-care-model (Asians and Latinos) [82] Preference for a medicine man over a family member for individuals with physiological problems (Navahos: indigenous) [53] Preference for cultural sensitivity within health services and for allowing older immigrants to die at home surrounded by relatives because of fear that outsiders would not provide the same level of care as family members [96]	Preference (Japanese Americans) for noncultural elements (e.g., transportation services, internet access) over Japanese culture-specific elements (e.g., Japanese cultural activities) when considering their choice of residency [67] Preference for Western health care providers and medical doctors over social workers (Navahos: indigenous). Does not indicate that traditional healers are not being used [53] Preference for the Traditional Case Management Model (New Approach 3) (Majority of African American, Latinos and White Western-European American (only half of the Chinese participants)) [84] Preference for Complete Control Over Service Areas (CLTC decisions, services and worker-related issues) [84] Preference for complete control over selecting the type of service (20% African American and 15% White Western-European American (5% Chinese and 5% Latinos)); preference for complete control over scheduling of their service [84]

#### Principles of housekeeping services.

The subcategory *housekeeping services* highlights the culture-specific expectations of care and support services among older migrants and people from minority ethnic groups. We identified preferences for high standards of good practice [60,65], defined as specific practices within one’s own culture, e.g., the importance of hygiene: *“I had to keep an eye on what home care we were doing; how we care for ourselves is not how the English would take care”* (Afro-Caribbean individual) [60]. With respect to care services, we identified a preference for Greek-speaking service providers [65] to overcome the language barrier, described by one individual as follows: *“Language in particular was seen as a key issue for some participants in light of their own limited English”*. Japanese Americans mentioned that they preferred cleanliness in the facility [64] because Japanese American families *“believed that cleanliness was a Japanese trait and Japanese American homes were cleaner. If the facility was clean and odorless, this was thought to enhance resident morale and symbolized the owner’s high regard and respect for the residents”*. Korean Americans also seem to have a clear idea of cleanliness. Korean Americans who preferred senior housing had a preference for senior housing exclusively for Korean Americans and support provided by Korean American home care program services [85].

#### Principles of medication.

The subcategory *medication* describes preferences for different ways of administering various forms of medicine. Traditional medicine has evolved over thousands of years, and various psychological and/or physical approaches (such as acupuncture and tai chi) and herbal products have been used to address health problems and improve quality of life.

***Herbal remedies***: Hispanic individuals in the USA preferred traditional herbal remedies, such as raw herbal tea, [58] and Korean Americans [69] preferred herbal medicines (han-yak is a traditional Korean medicine) and other remedies, such as acupuncture, moxibustion, and cupping. One study revealed that Indian immigrants in Canada preferred a mix of traditional remedies supplemented, if possible, by elective oral health care in India and by emergency dental care in Canada [71].

***Religion***: The included studies revealed that Black Americans [101] and African Americans [66] preferred to use prayers or faith for pain *management/care: “only God can heal”*. African Americans preferred faith-based resources with respect to attitudes about mental health care: *“African Americans in particular preferred to consult a member of the clergy or a primary care physician and to obtain care in medical and church locations.”* [59]

***Self-medication***: Another study [54] revealed that ethnic Chinese in Australia preferred self-medication when they experienced minor illnesses. Additionally, when visiting a general practitioner, they preferred Cantonese-speaking staff, although this preference depended on the severity of their illness.

#### Principles of leisure activities and principles of food.

Leisure activities refer to activities in which individuals spend their free time outside their mandatory activities (such as work, school, and sleep). These activities are based on open consciousness, free choice, and self-determination and include reading, sports, climbing, social activities, chatting, and shopping. Participation in leisure activities can improve the physical and mental health of people and their ability to regulate their body and mind, reducing life stress and ensuring a pleasant experience [102]. Leisure is central to the processes of home-making, identity building, and meaning-making; social belonging is also expressed through interactions with people with the same cultural or migratory background [103]. Studies focusing on leisure activities for immigrants consider spaces for immigrants that are separate from those of the general population owing to various constraints, including inadequate language skills, a lack of knowledge of the host society, social isolation, and cultural differences, that these individuals have experienced.

In terms of the relationships between ethnic food and a sense of belonging in immigrant life, studies have shown that eating food “*like at home*” (i.e., ethnic food) can successfully reduce the sense of loss that immigrants may feel and may enhance their sense of belonging to their host society [104]. The consumption of ethnic foods is also described as a vital strategy that people with migration a background adopt to connect their previous life with their present life [105]. Furthermore, people with a migration background may attach their sense of belonging to a particular place or space by using specific foods as linkages or references, which can further alleviate and decrease their feelings of nostalgia [106].

People with a migration background refer to foods from their home country as “*comfort foods*” in their new life, since these foods have positive psychological effects and enhance well-being [107]. Furthermore, Schermuly and Forbes-Mewett reported that many migrants share traditional or ethnic foods from their country of origin with friends, neighbors, or the community in their new home country as a useful strategy for expanding their social networks and interpersonal relationships and managing new friendships [108].

Eleven studies identified preferences for leisure activities with a tendency toward the culture of origin (Table 5). Older Pakistani individuals in Norway seem to prefer Pakistani TV [52,96], whereas older Korean Americans prefer Korean cable TV [69]. Germans in Australia appear to adapt to the Australian way of life and do not show high interest in ethno-specific services. However, preferences for German cultural traditions, such as celebrating Christmas Eve (rather than Christmas Day) or buying groceries at a local German delicatessen store, are still valued. Non-

**Table 5:** Identified preferences for leisure activities and traditional food.

Leisure activity		Food	
***Tendency to country of origin:***	***Tendency to immigrated country:***	***Tendency to country of origin:***	***Tendency to immigrated country:***
Preference for Pakistani [52] and Korean [69] TV activities non-Latino Black individuals show a preference for activities other than bingo, e.g., faith-based activities, religion, reading, arts [62] Korean Americans prefer church services conducted by Korean pastors [69] Germans in Australia show a preference for German traditions: e.g., visiting German delicatessens and celebrating Christmas Eve [80] Preference for having their “own work” to not feel useless while living in a facility [influenced by the Japanese culture, 64] Preference for Korean cultural activities: reading a Korean newspaper, playing *hwatu* and *yut* (a Korean card game), participating in karaoke with Korean words on a large television screen, having tea, participating in cooking activities featuring Korean cuisine, and gardening and growing plants; Korean female residents tended to prefer doing their own laundry [69] Preference for outdoor and/or off-site activities: attending Korean-style barbecue parties, eating at Korean restaurants, and shopping at Korean markets [69] For Sámi residents, it is important to have activities that include elements from their Sámi way of living [94] Preference for Finnish cultural preservation, as seen in church services with singing [98]	No preference for Japanese TV [64]	Preference for culturally specific, traditional or religious food [52,57,60,61,64,69,75,77,78,86,89,90,92,93,98] Preference for traditional Sami food and the wish for assistance in preparing Sami food (regardless of whether elders were close to the Sami culture) [76] Preference for giving food primarily as a means of reciprocating for care (language of food as a traditional way of socializing) [78,91]	
		Preference for typical Cuban food – “helps to self-identify as Cuban” (*Acculturation*: “Cubans have added some American food to their diets, and they have modified their meal times and meal patterns” [79])	

Latino Black individuals from the USA prefer activities other than bingo [*“for most of them, they think Bingo is the bomb, but for our ethnicity, it’s—you know, we’re involved in much more of the arts”]* that fit their culture (such as faith-based activities, arts and reading) [62]. Older people from culturally and linguistically diverse backgrounds living in nursing homes in Australia prefer enjoyable meaningful engagement, such as cultural events and celebrations as well as music, cooking, games, traditions and holidays, contact with animals, attending church, and events that celebrate people’s diverse cultures. Furthermore, ethno-specific nursing homes, by connecting with community organizations for leisure activities, were able to meet residents’ preferences for maintaining the religious activities they had enjoyed throughout their lives [91,99]. Xiao et al. reported that older Finnish residents in Sweden actively preserved familiar Finnish customs through participation in activities organized by the Finnish parish (church services) and the Sweden-Finnish Association (choir concerts), with singing being a cherished practice [98].

Eun-Hee Lee [69] analyzed three case studies of Korean Americans living in nursing homes and investigated how culturally appropriate environments in nursing homes are for frail older people from ethnic minority groups in the U.S. The study revealed various preferences for indoor and outdoor leisure activities expressed by Korean Americans: reading a Korean newspaper, playing hwatu and yut (a Korean card game), participating in karaoke with Korean words on a large television screen, having tea, participating in cooking activities featuring Korean cuisine, and gardening and growing plants. Korean female residents liked doing their own laundry (indoors). Older Korean Americans preferred outdoor and/or offsite activities: attending Korean-style barbecue parties, eating at Korean restaurants, and shopping at Korean markets. Korean Americans also expressed their preferences for church services conducted by Korean pastors [65]. Hikoyeda et al. reported that older Japanese women living in the USA did not prefer Japanese television while living in a facility. However, Japanese older women voiced a preference for having their *“own work*” because they did not want to feel useless, which mirrored their own cultural heritage understanding [64].

Eighteen of the 50 included studies reported a preference for culturally specific and traditional dishes and religious food. Food must be culturally familiar and should meet any religious requirements as well as the dietary requirements of the health condition of the individual being cared for. Ness et al. identified a preference for traditional Sami food and, furthermore, the wish for assistance in preparing Sami food (regardless of whether the elders were close to Sami culture or not) [76]. Older Cubans in the USA indicate a strong preference for typical Cuban food, e.g., white rice and black beans, pork and pig roast, which helps them “*identify as Cuban*”. However, Cubans have also added some American foods to their meal times and meal patterns as a process of *acculturation* [79]. Food sharing is a traditional way of socializing in the Philippines. Older Filipino people in Canada prefer to give food preliminarily as a means of reciprocating for care [109]. Older Asian Americans who reside in care facilities have a strong preference for traditional Asian food. However, their preferences for traditional Asian food often goes unmet; thus, family members may provide meals when the facility’s offerings fail to meet the dietary needs of their older relatives [93]. People from culturally and linguistically diverse backgrounds in Australia associated the availability and preparation of traditional food with health, enjoyment, and engagement [91]. In their systematic literature review, Lillekroken et al. reported that older immigrants have specific food habits and meal preferences after immigration. Furthermore, older immigrants see traditional food as a part of their identity. For example, across all cultures, women tend to hold significant food-related roles associated with providing food for their families as wives and mothers. Moreover, older immigrants’ new food habits and food culture adaptations are reported to be influenced by the length of residency, age, access to traditional foods, and changes in living environments [92].

### Preferences within the professionals category

The main category *professionals* addresses identified preferences regarding nursing care procedures and services as well as health care assistance provided for older migrants and people from minority ethnic groups. Here, professionals refer to health care professionals, physicians, nurses, care staff, therapists, and other licensed nursing personnel.

#### Sensitive to other cultures.

To be sensitive to other cultures means being aware that cultural differences and similarities among different people exist without assigning them a value—positive or negative, better or worse, right or wrong. However, being sensitive to other cultures involves a set of personal skills that allow a person to understand and learn about people with cultural backgrounds different from their own. Hefele et al. identified a preference for staff that are sensitive to Latino culture [62]. Furthermore, Pasco et al. reported a preference for special ways of touching, which indicate a nurse’s respect for the individual being cared for:

*“Filipino participants had different terms for the way they were touched: haplos was a tender touch, hipo was a soothing touch to ease pain, salat was palpation, himas was the touch with a distinct pressure as used in massage, and hilot was equated to bone alignment or physiotherapy. Patients distinguished whether a nurse’s touch was gentle or rough and whether it reflected the nurse’s respect for the patient”* [78].

Another identified spiritual preference was the use of gaze, which means looking straight at a nurse when receiving instructions (and vice versa) [78].

#### Use of an interpreter.

Giuntoli et al. reported that older migrants in the UK who were not fluent in or able to speak English preferred interpreters to ensure effective communication. The removal of language barriers is seen as the first step toward fostering trust and a greater understanding between older migrants and those who provide care [60]. Xiao et al. reported that older residents in Australia with a migration background expressed a preference for culturally competent bilingual interpreters to bridge language and cultural gaps in their care [99].

#### Specific perspectives on ethnicity.

The subcategory *ethnicity* captures the preferences of people with a migration background for nursing staff, care professionals, and other medical staff with a similar/the same cultural background.

For ethnic minority residents living in Australia, meeting their care needs and thus their preferences depends on staff members’ cultural competence and willingness to overcome related challenges [98]. Chinese Americans preferred having a doctor of the same race [50]; however, there was no evidence of whether there was a preference for a doctor to have a migration history or to share the same cultural experiences. Older Pakistani immigrants in Norway preferred home care professionals of the same ethnicity [52]. In contrast, Chinese elders in Canada preferred (being treated by) trained Western doctors rather than traditional Chinese practitioners and preferred receiving treatment within their own culture by Chinese staff who spoke the same language as they did [56]. Ibrahim et al. studied the preferences of African Americans who were receiving care and did not find a clear preference for receiving care from a physician of the same ethnicity. Only 10% of the study population showed a preference for receiving care from a physician of the same ethnicity [66].

Older Korean Americans showed a significant preference for having staff members of the same ethnicity and expressed a preference for staff members with the same cultural background [85]. South Sami, an indigenous population in Sweden, seemed to prefer receiving care from Sami caregivers “*because of the risk that they [the patient] might go back to their first language if they suffer from dementia*”. However, when patients did not speak Sami themselves or were bilingual and/or had adopted Swedish culture, no preference for a Sami caregiver was identified [76]. Korean American elders showed a preference for having staff who shared the same ethnicity and the same cultural background because they experienced difficulties receiving appropriate care from non-Korean staff [69]. In a qualitative study conducted by Pasco et al., older Filipino Canadian participants preferred to be cared for by a nurse from their own culture so that they could converse in their own language “*because language can also include facial expressions, gestures, speech intonation, volume, and colloquialisms, and these can be misinterpreted in across-cultural context”* [78]. Specifically, Pasco et al. reported that Filipino Canadians preferred having a nurse who was *Hindi ibang tao* (“*one of us*”)—to share a common identity and experiences, e.g., being an immigrant, having a similar family role, or spending time with the patient and using the language of words, touch, gaze and needs, to disclose needs [78].

#### Native and/or bilingual language.

To meet the needs of individuals in care, health care systems and providers need to be aware of, and responsive to, patients’ cultural perspectives and backgrounds. The concepts of cultural competence and patient-centered care intersect in meaningful ways. Patient and family preferences, values, cultural traditions, language, and socioeconomic conditions are respected. The subcategory *language* shows findings of identified preferences with respect to different language experiences.

***Preference for the same language***: In their study, Hurley et al. reported that older Greeks prefer to receive care from Greek-specific services because of fewer language barriers [65]. MacEntee et al. reported that Punjabispeaking South Asian immigrants in Canada preferred having a dentist from the Punjabi community because it was easier to explain the problem (avoiding language barriers) [71]. In 1998, G. Netto reported that minority ethnic carers of older people in the UK opposed sitter services and preferred the sitter to speak the same language [77]. Individuals from Turkish, Moroccan, and Surinamese minorities in the Netherlands seemed to prefer home care providers who spoke their language [88]. South Sami individuals (the indigenous population) in Sweden preferred having care providers who spoke South Sami while receiving care in a nursing home, mainly because of the provision of a better understanding of language and culture. Ness et al. did not identify a preference for the same language when participants did not speak Sami themselves or were bilingual (in this case, they spoke Swedish). The reasons were, among other things, rejecting one’s South Sami background or adopting Swedish culture [76]. Rhodes et al. studied Bangladeshi immigrants in the UK and did not identify a clear preference for having a Bengali-speaking doctor. Female patients preferred having an English-speaking nurse to a Bengali-speaking doctor *“because the nurse was a woman and also because she was more approachable and prepared to try to listen to their concerns”* [81]. Chinese Canadian patients and their family caregivers preferred staff, therapists and health care professionals who spoke Chinese [90]. Chinese immigrants in Australia preferred receiving care from Cantonese-speaking general practitioners [54].

When interacting with staff in nursing homes, Korean American elders indicated a preference for staff who spoke the same language: *“Korean residents expressed that they had difficulties receiving appropriate care from non-Korean staff”* [69]. However, older people with migration backgrounds also had preferences for the ethnicity of staff. Lee (2012) identified a preference for Korean staff: *“The participants expressed the need for more staff from the same cultural and linguistic background to increase the quality of care and life of Korean American residents. A great majority of Korean American residents had no or little ability to speak and understand English. Therefore, they often experienced communication failures with non-Korean staff, particularly nursing aides. The communication issue between Korean residents and the staff was one of the most important considerations regarding the quality of care and life.”* [69]

In their qualitative study, Rhodes et al. reported that Bangladeshi immigrants in the UK strongly preferred that children/other family members accompany them to appointments because of strong language barriers [81]. Older people from culturally and linguistically diverse backgrounds in Australia preferred to communicate in their primary language and staff who spoke non-English languages in residential care facilities [83,91,99] so that they could express their preferences in their native language [98]. Permanent aged care facility residents born in a nonmain English-speaking country preferred communicating in a language other than English. These findings indicated that non-English-speaking residents in aged care settings face multifaceted experiences of isolation. While same-language support is often perceived as a remedy, its effectiveness is frequently compromised. This breakdown occurs either when a resident is the sole speaker of their language or when cultural and socioeconomic disparities endure despite shared linguistic backgrounds. Consequently, the risk of social isolation persists, even with language-based assistance, particularly for residents from diverse cultural origins within a language group [94]. Xiao et al. conducted a meta-synthesis of qualitative research and reported that using residents’ native languages significantly improved the efficiency of care and daily life. Finnish seniors preferred nursing homes with Finnish-speaking staff, primarily because of concerns about dementia-related language loss. Native language communication significantly improved care and daily life for these residents [98].

***Preference for bilingual staff:*** We found only one study in which a preference for bilingual staff was mentioned (in addition to the Sami-Swedish population). Hefele et al. reported that older Latino people preferred receiving care in a nursing home with bilingual or Latino staff [62].

***Process of acculturation***: One study revealed that older German Australians successfully adapted to life in Australia, largely because of their high proficiency in English. This linguistic ability enabled them to navigate services effectively, avoiding the language barriers commonly faced by many older migrants. Their lower interest in ethno-specific services as a result implied that they prefer mainstream offerings, indicating strong integration into the broader Australian community. This adaptation may reflect their desire for broader social engagement and access to resources that align more closely with their everyday lives [80]. Furthermore, Shrestha et al. reported that the preference for nursing homes emerged only when severe immobility occurred. However, caregivers, who had adapted to Norwegian norms, considered residing in nursing homes in the future, anticipating fewer challenges than their parents faced [97].

#### Biological sex.

We identified six studies in which older Chinese Americans [110], Pakistani women in Norway [52,97], Pakistani and Bangladeshi immigrants in the UK, African Americans [66], and Asian immigrants in the UK [77] expressed a preference for staff or professionals of the same sex as themselves. Arora et al. [52] reported that the biological sex of professionals became relevant when care required physical touch or any form of intimate care.

### Preferences within the living with others category

#### Individual living arrangements.

The terms “living arrangements” and “coresidential arrangements” are used interchangeably to refer to the structure of the household in which older individuals reside. When an older individual resides with at least one child, the term “coresidence” is used. Unless otherwise noted, when an older individual resides with a spouse but no child or was unmarried, the term “living alone” is used [111]. Living arrangements varied across older adults and included living alone, living with children, and living in a nursing home or residential community. This subcategory identifies the preferences of older people from different cultural backgrounds with respect to their preferred living arrangements.

Greek elders in Australia preferred having family around when receiving formal care and strongly preferred receiving informal assistance from family (family members or informal carers) [112].

Issei women (Japanese Canadians) expressed interest in using services in combination with a preference for receiving care from children. Individuals who identified as Japanese Canadians chose to receive institutional care; those identified as Japanese depended on their children. Those who identified as Canadian Japanese preferred to use formal services, and one-third of these individuals preferred to receive residential care. Participants who identified as Japanese preferred to move into their children’s homes and depend on their children, whereas those who identified as Japanese Canadian preferred to move into institutional settings and receive institutional care. People who identified as Canadian Japanese indicated a preference for purchasing services and living in an apartment or remaining in their present situation. In addition, Canadian Japanese older people preferred easily purchased services to move into an accessory apartment [72].

Older Pakistani immigrant women in Norway perceived professional home care services to be more acceptable than residential care homes were [52]. Overall, older Pakistani individuals express a preference for remaining in their homes and living together with their adult children [97]. Older immigrants from diverse backgrounds preferred coresidence with their family members [96].

Asian Indians in the USA preferred living arrangements in which they lived near their children rather than with them. They did not prefer to receive institutional care; instead, they preferred to return to India [113].

For Korean Americans, the type of preference differed depending on the type of disease and the need for care. They preferred mixed arrangements (e.g., home-based health care and care from professionals at children’s homes) in the hip fracture scenario. In the stroke scenario, more than half of the older Korean American study participants preferred formal care arrangements (formal caregivers and care locations), whereas only ¼ of the participants preferred informal care arrangements, and 1/5 of the older Korean American participants preferred mixed arrangements. Older Korean Americans preferred all informal or mixed-care arrangements for short-term care in the hip fracture scenario, but they preferred all formal care arrangements in stroke scenarios. J.W. Min identified different influencing factors, such as traditional values, better self-health status, higher education levels, and sex [73].

Older Korean Americans preferred living independently in senior housing or coresiding with their adult children over living in a nursing home if they became bedridden. Older Korean Americans may not want to live in a nursing home, but changing circumstances may make it unavoidable, e.g., because of caregiver burden and when nursing home services are free of charge for low-income individuals [85].

Montayre et al. identified two major themes from the analysis of interview data from older Filipino immigrants in New Zealand. The first theme, “preferred living and care arrangements,” involves older Filipinos’ preferred plans for future living and care arrangements when they are no longer able to function independently in their own homes. The second theme describes issues such as cognitive ability, safety, and independence in maintaining one’s own cultural beliefs and practices. Most older Filipino immigrants preferred residential elderly care when they were no longer able to live independently but lacked knowledge about elderly care facilities. Some older Filipino immigrants mentioned having an intention to live with family members but also realized that coresidence with adult children may not be possible, particularly on a long-term basis; they considered other options, such as living in elderly care facilities, when coresidence was implausible, even if this was not the best option for living and care arrangements later in life. A few older Filipino immigrants in New Zealand preferred to return to the Philippines if living with family was not possible [75].

Great Lake American Indians preferred to receive noninstitutional home-based care and to remain in their own homes, although acculturation levels influenced these choices [55].

Older Japanese American women preferred private accommodations rather than shared rooms while living in residential elderly care facilities because these facilities give residents a sense of freedom and control [64].

Older African American individuals viewed living in a nursing home as an acceptable alternative for care when family care was not possible [68].

#### Organizing informal care.

As noted above, migrants and people from diverse minority ethnic groups have diverse cultural backgrounds and reduced access to care. Personal and social values, culture, and knowledge about health care options influence individuals’ health care seeking behavior. Migrants born in countries outside Europe provide care to family members to a much greater extent than European-born people do. One explanation may be that differences in caregiving patterns may reflect that people born outside Europe, compared with European-born people, have different attitudes toward caring for their relatives. In addition, many migrants face different challenges regarding access to and use of primary care [114]. The following results reveal preferences regarding informal caregiving across diverse migrant populations and minority ethnic groups.

M. Polacsek investigated the support needs of older German individuals in Australia, and the findings revealed no preference for family caregiving [115]. Japanese Americans also showed no preference for family caregiving in long-term care planning [67]. Older Chinese immigrants in the USA showed a preference for receiving care from children following the cultural tradition of showing respect by caring for elderly family members [116].

Older Pakistani immigrants in Norway prefer to receive care from family members, mainly children, in old age [52]. English-speaking and Arabic-speaking Arab-American immigrants in the USA have different expectations of children with respect to care: being a burden on children and believing that children have an obligation to care for their elderly parents, respectively. There is also a critical difference between fluent English speakers and those who prefer to speak Arabic, who differentiate themselves from ‘Americans’, often by characterizing children’s obligations as an act of ‘compassion’. These observations demonstrate that there is no clear agreement on the expected role of children in providing care and support in times of need. While some participants strongly felt that caregiving “is an obligation, others referred to the difficulties the responsibility created” [49]. Filipino Canadians preferred to be cared for by a nurse from their own culture but also preferred that their family members provide assistance and care [78]. African Americans, especially Latinos, expressed community-oriented, collectivistic views of caring for older individuals and preferred to receive care from family caregivers (spouses or children). Furthermore, African Americans preferred to have female family caregivers [82]. Giuntoli et al. investigated the experience and expectations of care and support among diverse older migrants in the UK. Polish immigrants in the UK preferred having their children take care of them and did not want any external help because “they had often developed a strong fear of ‘officers’ as a consequence of their experiences during the Second World War” [60].

Issei (Japanese Canadians) who immigrated before 1950 strongly preferred to receive care from children, *“usually a female member of the family who is selected to care for older people”*, or family members, as it is *“not a boy’s job to take care of parents”*. Whereas Nisei, second-generation Japanese Canadians or their children, preferred purchasing services or depending on their spouses for care, where men preferred to receive care from their wives [72]. Mexican Americans (Latinos) in the USA strongly preferred family caregiving and indicated that children should be responsible for elder care [73]. Older Greek individuals in Australia strongly preferred receiving informal assistance from family. Furthermore, they preferred to have family around to support them when they received formal care [65]. Bangladeshi immigrants in the UK preferred to rely on their relatives, even when alternatives were available, e.g., as interpreters [81].

Suurmond et al. (2016) reported that both people from minority ethnic groups, consisting of Turkish, Moroccan and Surinamese immigrants, and older Dutch individuals, “were aware that their needs could be addressed by a formal home care institution, but they preferred that their family members take care of their needs”. Furthermore, Dutch participants expressed a preference for women to take care of the needs of elderly family members. Older people from the three different minority ethnic groups and ethnic Dutch older people had comparable preferences for receiving home-based care from family members. However, older people from minority ethnic groups considered formal home care only when family members could no longer cope [88]. Min (2009) reported that 55% of older Mexican-American individuals preferred to rely on a formal/professional helper, whereas 45% would turn to informal caregivers or helpers when faced with care needs following hip fracture. In comparison, older non-Latino White individuals preferred having formal/professional help (83.3%).

Older Mexican-American individuals strongly preferred family caregiving and believed that children should be responsible for elderly care (MA) [73]. Sudha (1999) reported that African Americans were more likely than White Americans to prefer family care. The results of the study revealed that older people and men preferred family care more. Higher socioeconomic status is associated with less preference for family care. Furthermore, men preferred family caregivers more. Education level also influences the preference for family caregiving [87]. Asian Indians in the USA show a preference for children as caregivers (older people) and for relying on family. In contrast, midlife adults no longer prefer that their families/children care for them [113].

With respect to primary caregivers, older Korean Americans show a preference for being cared for by a female family member, e.g., daughter or daughter-in-law [85]. Johnson et al. (2001) reported that older ethnic individuals “cope with their need of assistance by relying predominantly on their families or on other informal sources, such as prayer, denial, displacement or worry”. Furthermore, family support is the preferred system and is commonly provided by adult children among Latino elders (particularly those not born in the U.S.). African Americans as well as older Pakistani living in Norway prefer support from spouses and adult children. If adult children may not be available, it is common for the social network to be molded to meet the needs of older members by including distant or nonrelatives [68,97]. Older Pakistani immigrants preferred to be cared for by their children, especially because of the moral obligation of caregiving. However, early childhood migrants appeared to note differences in caregiving norms between themselves and their European-raised children, leading them to anticipate reduced reliance on their offspring in old age, reflecting their own cultural adaptation [96].

#### Ethnic identity within the community.

Living in a community means experiencing a feeling of belonging and togetherness and having the chance to meet new people. Community members care for each other while living in the community together. For all members, it is important to belong to a community—as individuals—and to be active within it. Living in the community and participating within a community are the responsibilities of every ‘community member’. Every community has its own beliefs, norms, and expectations, and as such, the community can be both empowered and disempowered.

Arabic immigrants in the USA prefer nursing homes “*just for Arabs, where they can eat Arabic food*” [49]. Older Pakistani immigrant women in Norway prefer to have a separate care home or a separate area within the building for Pakistani, or Muslim, older people. They show a preference for sex-divided care homes (separate care for women) [52]. Great Lakes American Indians expressed a need for facilities that are managed and owned by Indians that are community-based and intergenerational, involving entire families, that would provide “*assisted living*” and home-based care and, most importantly, represent the traditions, beliefs and spirituality of Native people [55]. Japanese Canadians prefer Japanese nursing homes [72].

Older Korean Americans reveal a preference for senior housing exclusively for Korean Americans with support provided by Korean American home care program services [85]. Older Chinese immigrants show a preference for adult day care and living in public housing with other Chinese-speaking older individuals [116]. Minority ethnic carers who care for older family members (with different migration backgrounds) have specific preferences for respite services. However, the concept of residential respite care was completely novel to all of the carers; the carers preferred that those whom they were looking after were in social contact with people of their own ethnicity outside their home. Carers’ preferences for the person they are caring for to be with others of the same ethnic origin “strongly suggest that they would welcome home respite services which would allow the older people to be cared for either in their own home or in the home of a family from the same minority ethnic group” [77].

Older Asian Indians in the USA preferred to join community-specific cultural groups for social activities [113]. Older Japanese Americans in the USA preferred residential and long-term care communities where a mixture of Japanese and non-Japanese individuals reside [67]. Older non-Latino Blacks and Latinos preferred living in nursing homes with residents of the same race/ethnicity (fit in the community) [62].

### Preferences within the environment category

The physical and social environments in which people are surrounded and live also affect health and well-being. The environment plays an important role in determining a person’s physical and mental capacity across different stages of life as well as into older age. Both older people and the environments in which they live are diverse, dynamic and changing, and through their interactions with each other, they have the potential to promote healthy aging [117]. The environment subcategory indicates the identified preferences regarding the cultural familiarity of the environment and the facility characteristics.

#### Cultural familiarity.

The choice of home significantly facilitates a more positive transition for older individuals in need of care. The global population is aging, and nursing homes are increasingly providing care to older people with multiple and complex needs. The move to long-term care in a care facility is always a challenge but can be managed more effectively by increasing awareness of the importance of familiarity, stability, and social capital in the lives of older people and their carers [118]. Furthermore, understanding cultural factors can help nursing staff gain new insight into the transition of older individuals to residential care facilities [119].

For example, Japanese American older women preferred Japanese-oriented residential care facilities for elderly individuals (RCFEs) [64]. Older Chinese immigrants in the USA preferred Chinese walking spaces in care facilities (outdoor Chinese pathways) [70]. Older Korean Americans preferred culturally tailored services (key factor of not preferring coresiding with children) [85]. Great Lake American Indians and the indigenous population in the USA preferred facilities that represent the traditions, beliefs, and spirituality of Native people [55].

#### Facility characteristics.

Phlix et al. noted the effects of the dynamic nature of age and aging and migration background on the subjective well-being of older migrants. The need to preserve one’s housing situation and environmental autonomy later in life is important. Furthermore, the mutual influence of subjective well-being and sense of home influences one’s personal impression of the home. The authors highlighted the intersection of age, migration, and housing with the subjective well-being and perception of home among older migrants. Furthermore, both material (i.e., housing) and immaterial (i.e., sense of home, age, and migration) factors influence older migrants’ subjective well-being [120]. Japanese American older women preferred Japanese or Japanese-oriented facilities, e.g., Japanese staff, Japanese food at least once a day, activities focused on Japanese culture, Japanese television, and a Japanese environment. Furthermore, they prefer “*home-like*” environments and smaller facilities with fewer residents [64]. Older Korean American individuals living in ethnic nursing homes preferred to have a house or bedroom facing south. They also preferred an “*Ondol Room*” (traditional Korean heating system = underfloor heating in Korean housing): *“I prefer an ondol room to a carpeted floor. The room is good for our back pain and suitable for our bodies”; “Almost all of them were using ibul or dam-yo instead of the bed quilts provided in the facility because they either felt the facility’s quilts were too thin or they preferred Korean-style bedclothes.”* [69].

Pakistani and Bangladeshi study participants stressed the importance of their identity as Muslims. For them, it was important to acknowledge the practices and behaviors related to their religion in service provision. Through culture- and religion-tailored service offerings, they intend to provide halal food, same-sex care staff, and prayer rooms in housing services [60].

## Discussion

To our knowledge, our scoping review provides the first comprehensive overview of the current care preferences of older migrants and minority ethnic groups with various care needs. Moreover, we explored the existing care preferences of older migrants and people from minority ethnic groups in different care settings and clusters and synthesized the different characteristics of existing care preferences. Our analysis, which involved 50 articles, revealed descriptive preference themes that were grouped into four categories: I) care practice; II) professionals; III) living with others; and IV) environment. These categories were further divided into 16 subcategories that captured the identified preferences (see Table 3). However, in this review, we identified a wide range of different populations with distinct migration backgrounds and diverse minority ethnic groups with various care needs in different care settings.

Ethnicity and religion, specifically in the migratory context, became important identity markers and can be subjectively used to define or express a sense of identity [127]. Migrants themselves constituted the arrival in the receiving country as a “total” event because it required a (re)construction of their own identity. When migrants leave their country of origin, they often lose their social status, family, and social networks. In the receiving country, migrants find themselves without a history. When facing the unknown, migrants may feel lost, alone, and without reference points in an unfamiliar society. As much as they aim to become integrated into the new society, migrants often remain strangers. Moreover, they often face distrust and hostility.

The harsh reality of exclusion differs from the idealized image of the receiving country as a place to improve one’s life, which originally drove migrants to leave their country of origin. The resulting disillusionment and nostalgia often contribute to idealizing the country of origin, which is in turn beautified through memory. However, if a migrant returns to their country of origin, the contrast between the ideal scenario and the reality reappears. To a certain extent, migrants live between idealization and disillusionment both in the receiving country and in the country of origin. As a result of this complex transition, their new reality is in between, at the borderland, and in transit. It becomes evident that the process set in motion when migrants leave their home country never fully comes to an end, generating a continuous state of not yet belonging “here” but no longer belonging “there.” [128]. The descriptive interpretation of the available literature shows numerous preferences within different categories. However, it is not sufficient to summarize preferences because different changes occur on the basis of various migration flows. Thus, according to the acculturation examples, the migration process clearly impacts a person’s identity. The question of how identity shapes preferences is not easy to answer because migration itself has a significant effect on care preferences, among other things.

When people migrate from one country, particularly the country in which they were born, to a new country, which may become their new home, they find themselves in a situation in which they compare how their life and practices were before to how they are now, illustrating that the new “culture is the way we do things around here” [121].

Duranti (1997) discussed several theories of culture that prioritize language, examining culture as a shared system of knowledge, a tool for communication, a means of mediation, a set of practices, and a framework for participation [122]. Gaps in knowledge and challenges in information delivery contribute to significant health disparities among diverse communities. Health information can help individuals understand and manage their health care. Different studies have shown that immigrants, people with lower socioeconomic status, and people who are uninsured are less likely to seek health information or, moreover, encounter negative information-seeking experiences, which can adversely affect information seeking as well as self-efficacy and health behaviors. Minority ethnic groups and migrant populations experience language barriers, health literacy issues, and cultural differences, which are potential reasons for gaps in information [123].

Language is a system of communication that consists of a set of sounds and written symbols that are used by the people of a particular country or region for talking or writing. For individuals who speak languages other than English at home, health and health care may be compromised by their difficulty in communicating their medical needs to providers who do not speak their preferred language [124–127]. Language barriers can also affect patients’ experiences of care and compliance with provider recommendations. Compared with English-preferring patients, non-English-preferring patients often report more problems with primary care provider communication, access to care, timeliness of care, and health plan customer service [128].

Various studies have addressed cultural identity at the group level [129,130]. Although culture often refers to a group dimension, the dynamic tension between the individual as a social player and the individual subject position within the group needs to be addressed. The question is how individuals deal with what they refer to as “culture” and how they conceptualize their understanding of their own cultural identity. These conceptualizations might be revealed through metaphors or what various studies refer to as metaphors of culture, and they can be important elements in a speaker’s individual identity constructions in discourse [131]. Identity is the outcome of the negotiation of personal given and already set conditions, social context, existing or newly emerging relationships, and institutional frameworks. Various studies have shown that migrants’ patterns of identification vary widely—from identification with the country of origin, religion, or mother tongue to identification with the receiving country, neither country, or both countries [132–135].

However, identity is a broad concept that has been defined differently across disciplines. The core mechanism of individual and collective identities lies between self-representation and social categorization. Individuals differentiate themselves by adopting different criteria that are shared by the members of a social group and by developing a sense of belonging to it. When a social group recognizes individuals’ belonging, a collective identity emerges [136]. Specific behaviors associated with embodied roles undoubtedly change over time and across space. As a result, the cognition, representation, and definition of identity also change. This shift becomes particularly clear during migration. It appears that individuals perceive identity as fluid and multiple. Identity is better described as something individuals “do” rather than something they “have,” as a process rather than a property [136]. With this review, we are one step closer to answering our original question about which preferences should be addressed with respect to migrants. We summarized the main trends in the literature.

Our findings emphasize that identity, cultural awareness, and acculturation are not only theoretical constructs but also represent critical gaps in current healthcare practice. Understanding how older migrants and members or minority ethnic groups perceive their identity and navigate processes of acculturation is essential for designing culturally responsive care. These aspects affect how much people trust, communicate with and engage in care. On the basis of the findings of this review, it becomes evident that healthcare professionals and institutions need to more consciously integrate cultural awareness and language preferences into everyday care. The results show that such aspects are closely linked to how older migrants and members of minority ethnic groups experience quality of care. Acknowledging individual cultural backgrounds, offering bilingual or culturally concordant staff, and encouraging reflective training on identity and diversity could therefore help translate person-centered principles into culturally competent practices and make care more inclusive.

The preliminary objective of this review was to identify the care preferences of international migrants with a legal status; therefore, we did not include populations such as refugees. Furthermore, there is a need to increase the awareness of migrant populations and their care preferences.

In this review, we identified a wide range of populations with a migration background and diverse minority ethnic groups with various care needs in different care settings. Personal and social values, culture, and knowledge about health options influence individuals’ health care seeking behaviors. As mentioned previously, migrants and people from different ethnic minority groups have various cultural backgrounds and often have limited access to care in the new home country. One explanation could be that differences in care patterns may occur due to people being born in countries with less sophisticated health care systems, resulting in different attitudes toward caring for their relatives or seeking professional care services. Many migrants face different challenges in accessing and using basic services [137]. Overall, the results from the literature review revealed that older migrants, regardless of their cultural background and new home country, have similar ideas regarding identified care preferences.

## Limitations

The main limitation of this scoping review is the current lack of homogeneous/constant use of the term “preferences” with respect to older migrants and people from minority ethnic groups, since a variety of terms and synonyms for “preferences” are used broadly.

Second, we focused exclusively on international migrants who were living in another country, different from where they or their (grand)parents (or ancestors) were born and who had a legal migration status [138] (e.g., dual citizenship) in the country to which they migrated. Furthermore, we focused only on international migrants with a legal migration status in the country in which they currently lived but not on other types of international migrants (i.e., return migrants and circular migrants).

In our review, we focused on international migrants with a legal status that provided access to a variety of care settings. We explored the care preferences of people (older migrants and/or people from minority ethnic groups) with various care needs across different care settings, without restrictions to specific countries.

For a better understanding, we clarified that undocumented migrants would not be excluded from our review, primarily because we anticipated that the reporting of migrant status could be weak in the identified studies. We need to trust that the researched population has been defined correctly in the included studies. Furthermore, we did not include individuals such as refugees. We did not include this population in our study because our preliminary interest was to identify the care preferences of international migrants with a legal status. We agree that refugees receive more services and rights than documented migrants do, but we believe that this population and their health care needs, as well as their care preferences, need to be researched in further studies. There is clear reporting of the differences among migrants, refugees, and asylum seekers in the identified studies.

Before we started our review, we performed a quick search and determined that in addition to the use of different synonyms for the term “preferences”, clear definitions of the populations were missing. We conclude that clear separation is often not possible because of the different terms and different understandings in different countries. We assumed that the terms ‘migrants’ and minority ethnic groups’ were useful umbrella terms for this review. With respect to ethnicity or race, there are a significant number of words, phrases and acronyms in the literature, which often change depending on the context. Language is continuously evolving; thus, understanding the meaning behind the terms used in research is important. In research studies, terms such as “migrant”, “immigrant”, “race” and “ethnicity” are typically used interchangeably. However, these terms have evolved in different ways and do not have the same meaning.

Another limitation of this review is the current lack of homogeneous/constant use of the term “preferences” for older migrants and people from minority ethnic groups, since various terms and synonyms for preferences are used broadly. Furthermore, we identified a lack of homogeneous/constant use of the terms used for migrants, and people from minority ethnic groups are used in diverse ways depending on the origin of the study.

The absence of standardized terminology for defining ‘older people’ and the variability in population nomenclature presented a significant methodological challenge. To preserve the integrity of the original study’s findings, we retained the terminology used in the extracted studies, despite the resulting terminological inconsistencies. However, the heterogeneity and inconsistency in the populations defined within the included literature reviews hindered a comprehensive extraction of all relevant demographic data from these studies regarding diverse populations.

The decision to exclude certain populations from extensive literature reviews was made on the basis of practical limitations. We acknowledge the potential for additional insights, but the thoroughness of the included studies provided a strong foundation for analyzing population migration. We believe that incorporating the excluded reviews would have resulted in negligible adjustments to our overall conclusions.

## Conclusion

The present results are preliminary and provide an initial overview of the current data landscape, highlighting the need for further critical reflection and more systematic research in this field. Our review revealed two key findings. First, preferences related to care are highly heterogeneous, reflecting diverse cultural values, personal biographies, and individual expectations that shape people’s attitudes toward care.

The migration experiences and experiences of ethnic minority groups further contribute to this heterogeneity. In addition to geographic relocation, migration involves psychological and social transitions that shape identity, belonging, and care expectations. Marginalization, discrimination, and acculturation also influence how individuals define autonomy, family involvement, and professional care. Consequently, care preferences among older migrants and minority ethnic groups reflect complex intersections between personal history, cultural norms, and social inclusion within the healthcare system.

This heterogeneity highlights that care cannot be approached only through standardized models applied in practice; rather, flexible, person-centered, and culturally sensitive strategies that acknowledge and respect identity and acculturation as well as individual care needs are needed.

Second, people with dementia and a history of migration—and those belonging to minority ethnic groups—and their specific care preferences in the nursing context have received limited systematic attention in healthcare research.

The lack of evidence limits the development of inclusive care practices and the ability of care systems to adequately respond to the needs of the increasingly diverse aging population. To address this gap, the DZNE Witten is conducting two projects with the aim of exploring and better understanding the nursing care preferences of older and long-term Russian late repatriates in need of care from the states of the former Soviet Union and of older people with a Turkish migrant background. These two groups are particularly relevant in the German and broader European context, as they represent some of the largest migration populations in Europe and have distinct migration histories, cultural identities, and care traditions. Russian repatriates often identify as ethnic Germans yet retain cultural and linguistic ties to their regions of origin, while many older people with Turkish migration backgrounds belong to the first generation of labor migrants and are now entering old age. Understanding their specific care preferences will help capture the diversity of migration experiences and inform more culturally sensitive care practices in Germany and other countries. These projects will contribute to a more differentiated understanding of culturally informed care preferences and support the development of more equitable and responsive care in the future.

In practical terms, understanding such preferences provides an essential foundation for improving culturally competent and person-centered care. The insights gained from this review can inform staff training, with a focus on cultural awareness, communication, and language sensitivity, and guide organizational practices. Integrating these perspectives into everyday care can help connect person-centered and culturally responsive care.

## Supporting information

S1 Table Study characteristics. (PDF)

S2 Table Search strategy in MEDLINE (via PubMed). (PDF)

S3 Table Search strategy in PsychInfo (via EBSCO). (PDF)

S4 Table Search strategy in CINAHL (via Ebsco). (PDF)

S5 Table Preferred Reporting Items for Systematic reviews and Meta-Analyses extension for Scoping Reviews (PRISMA-ScR) Checklist. (PDF)

S6 Table Supplementary Table 3: Summary of terms used in the included studies. (PDF)
