# Supplementary material for: Care preferences of older migrants and minority ethnic groups with various care needs: A scoping review
Source: PLoS One. 2026 Jan 23;21(1):e0341147. doi: 10.1371/journal.pone.0341147 (PMC12829939; doi:10.1371/journal.pone.0341147)
Supplement: S1 Table — (PDF) [file pone.0341147.s001.pdf]

Supplementary Table 1: Study characteristics

Reference	Country & study design	Study population & Setting	Self or proxy reported	Preference category
▪ Ajrouch, Kristine J. 2005	▪ United States<br>▪ Qualitative design - focus group interviews	▪ Arab-American immigrants<br>▪ Age: 60 years and older<br>▪ Sex: female N=19; male N=23<br>▪ Setting: aging experience and social support arrangements	▪ Self-reported	▪ **Facility Character:** Nursing home placements<br>▪ **Informal Care:** Expectations of children to give care
▪ Alley, Maxwell C., et al. 2016	▪ United States<br>▪ Quantitative design - customized, multidisciplinary paper survey	▪ Chinese (N=26) and White Americans (Caucasian) (N=42)<br>▪ Age: Chinese 73±9; WA 61±11<br>▪ Sex female: Chinese = 38%; WA = 50%<br>▪ Setting: Ethnic Barriers to Utilization of Total Joint Arthroplasty	▪ Self-reported	▪ **Gender:** Preference for doctor of the same race (Chinese)
▪ Aroian, Karen J., et al. 2005	▪ United States<br>▪ Qualitative design - semi-structured, open-ended interviews (individual or in focus groups)	▪ Older Chinese immigrants (N=27); Chines adult care giving children (N=11); health and social service provider (N=12)<br>▪ Age (median): Elderly = 78; Adult children = 44<br>▪ Sex: female Elderly = 63%; female adult children = 63,6%<br>▪ Setting: service use among Chinese elders	▪ Self-reported	▪ **Informal Care:** preference for children to take care of the elderly<br>▪ **Ethnicity:** public housing with other Chinese-speaking elderly<br>▪ **Medicine:** elders preferred Western over traditional medicine, depending on the nature of the health problem
▪ Arora S., et al. 2020	▪ Norway<br>▪ Qualitative design – 16 semi-structured interviews [in Urdu or Punjabi] and one focus group discussion	▪ Older Pakistani immigrant women (N=24)<br>▪ Age: 48 – 81<br>▪ Sex: female<br>▪ Setting= Older Pakistani women's healthcare access	▪ Self-reported	▪ **Informal Care:** family, mainly children<br>▪ **Care service models:** professional home care services<br>▪ **Ethnicity:** same ethnicity of the professional<br>▪ **Gender:** same sex of the professional<br>▪ **Facility character:** gender-divided care home (separate one for women).<br>▪ **Food:** halal food<br>▪ **Activities:** Pakistani TV
▪ Battistone M.L., et al. 1998	▪ United States<br>▪ Qualitative design – face-to-face interviews	▪ American Indians (in Navaho reservation) (total N=48; older N=27; younger N=21)<br>▪ Age: mean 61.5 (older N=27); mean 23 (younger N=21))<br>▪ Sex: male = 30%; female = 70%	▪ Self-reported	▪ **Care Service Models:** preference for Western health care providers

Reference	Country & study design	Study population & Setting	Self or proxy reported	Preference category
		▪ Setting: diagnosed physical and emotional illness; western vs. traditional health care providers		
▪ Chan Y. F., et al. 1997	▪ Australia<br>▪ Qualitative design – focus groups interviews [8 FG] (in Cantonese or either Mandarin)	▪ Chinese migrants (N=51)<br>▪ Age: 25 – 54 (N=27); 55 – 80 (N=24)<br>▪ Sex elderly: female N=18, male N=6<br>▪ Setting: use of health services	▪ Self-reported	▪ **Ethnicity:** Cantonese-speaking Genera practitioner
▪ Chapleski E.E., et al. 2003	▪ United States<br>▪ Qualitative design – face-to-face interviews	▪ Great Lakes American Indians: parent cohort N=252, middle aged children cohort N=74<br>▪ Age: parent cohort mean 69; middle aged children cohort mean 43<br>▪ Sex: parent cohort female = 63.8%, middle aged children cohort female = 60.8%<br>▪ Setting: future long-term care preferences	▪ Self-reported	▪ **Individual living arrangements:** remain on their own homes.<br>▪ **Cultural familiarity:** facilities representing traditions
▪ Chappell N. L., et al. 1998	▪ Canada<br>▪ Qualitative design – face-to-face interviews (77,2% in Cantonese)	▪ Chines elders N=1080<br>▪ Age: 76 (median)<br>▪ Sex: female = 63,6%; male = 36,4%<br>▪ Setting: health service utilization	▪ Self-reported	▪ **Medicine:** western over Chinese medicine<br>▪ **Ethnicity:** western trained doctors<br>▪ **Language:** Chinese staff<br>▪ **Care Service Models:** traditional Chinese care
▪ Dickson V. V., et al. 2013	▪ United States<br>▪ Qualitative design – interviews / Mixed-methods approach	▪ Ethnic Minority Black Population (N=30)<br>▪ Age: median 61.5<br>▪ Sex: male = 60%<br>▪ Setting: heart-failure self-care	▪ Self-reported	▪ **Food:** traditional food<br>▪ **Medication:** use of faith
▪ Dole E. J., et al. 2000	▪ United States<br>▪ Quantitative design – cross-sectional interviewer-administrated survey	▪ Hispanics (N= 84) and Non-Hispanic Whites (N=102) (N total =186)<br>▪ Age: 65 and older<br>▪ Sex: female Hispanics N=50; female NHW N=55<br>▪ Setting: established patients at the clinic health center	▪ Self-reported	▪ **Medication:** use herbal remedies
▪ Dupree L. W., et al. 2005	▪ United States	▪ African Americans (AA) (N=510) and White Americans (WA)	▪ Self-reported	▪ **Medication:** faith-based resource

Reference	Country & study design	Study population & Setting	Self or proxy reported	Preference category
	▪ Quantitative design - Self-administered surveys	(Caucasian) (N=216) (N total = 726)<br>▪ Age: total = 63.84 (mean); AA = 62.19 (mean); WA = 67.74 (mean)<br>▪ Sex: female (total) N= 454; female AA N= 320; female WA N=134<br>▪ Setting: 40 faith-based, health, community, and senior settings		
▪ Gaviola M.A., et al. 2024	▪ Australia<br>▪ Scoping review	▪ Chinese ethnicity, Finnish, Greek, Indian, Iranian, Italian, Japanese, Korean, Latvian, Mexican-American, and Indigenous peoples<br>▪ Age: no information<br>▪ Sex: no information<br>▪ Setting: dementia care for people from culturally and linguistically diverse background in nursing homes	▪ Self- and proxy-reported	▪ **Traditional food:** Availability and preparation of traditional food were associated with health, enjoyment, and engagement<br>▪ **Activities:** preference for enjoyable, culturally significant, and life-long activities facilitate meaningful engagement (music and singing along, cooking, games, traditions and holidays, contact with animals, going to church, and events that celebrate people's diverse cultures)<br>▪ **Language:** communication in their primary language
▪ Giuntoli G., et al. 2012	▪ United Kingdom<br>▪ Qualitative design – 1st phase: 21 focus group interviews (12 with elderly and 5 with carers); 2nd phase: 53 in-depth interviews with 38 older people and 15 carers	▪ Eight migrant communities; total N=167<br>- White British N=37<br>- Pakistani N=34<br>- Italian N=21<br>- Bangladeshi N=19<br>- Polish N=15<br>- Indian N=13<br>- Ukrainian N=12<br>- Hungarian N=9<br>- African Caribbean N=7<br>▪ Age: 60 and older N=129; >60 N=33<br>▪ Sex: female N=126<br>▪ Setting: care and support service user	▪ Self-reported	▪ **Informal Care:** White British: preference for family<br>Polish immigrants: preference for children<br>▪ **Gender:** Pakistani and Bangladeshi: staff of same sex<br>▪ **Interpreter:** interpreter to link effective communication<br>▪ **Facility characteristics:** praying rooms in housing service<br>▪ **House keeping services:** specific practices within the own culture<br>▪ **Food:** Pakistani and Bangladeshi preference for halal food [Muslims]
▪ Hanssen I., et al. 2016	▪ South Africa and Norway<br>▪ Qualitative design – indepth interviews	▪ ethnic Norwegians (in South Africa) and the Sami (in Norway) [indigenous populations]<br>▪ Interview participants:	▪ proxy-reported (family members and nurses)	▪ **Food:** traditional dishes

Reference	Country & study design	Study population & Setting	Self or proxy reported	Preference category
		nurses and family members total N=45<br>- Sami town setting: nursing staff N=9 and family members N=8<br>- Bergen setting: nursing staff N=3<br>- Tshwane setting: nursing staff N=19 and family members N=18<br>▪ Setting: nursing homes (dementia care)		
▪ Hefele J.G., et al. 2016	▪ United States<br>▪ Qualitative design – focus groups (N=11) and interviews (N=30)	▪ Non-Latino White (W) (N=27), non-Latino Black (B) (N=38) and Latino (L) (N=40) total N=105<br>▪ Age: 66.1 (mean) (range 42-89) W: 66.4; B: 65.4; L: 66.4<br>▪ Sex: female N=75.2 W: 85.2; B: 81.6; L: 65.2<br>▪ Setting: Nursing homes (experience/concerns)	▪ Self-reported	▪ **Ethnic Identity within the Community:** nursing homes with residents the same race/ethnicity (fit in the community)<br>▪ **Leisure activities:** preference for other activities than bingo, e.g. God, religion, reading, arts<br>▪ **Sensitive to other cultures:** staff that are sensitive to Latinos culture<br>▪ **Language:** NH with bilingual or Latino staff
▪ Hikoyeda N., et al. 2001	▪ United States<br>▪ Qualitative design – bilingual interviews (N=57) and unobtrusive observation	▪ Japanese American women N=26 and family member or legal representative N=31<br>▪ Age: 88 (average); Age family member: 65 (average)<br>▪ Sex: female (all)<br>▪ Setting: Japanese-oriented and non-Japanese residential care facilities for the elderly (RCFEs)	▪ Self- and proxy-reported	▪ **Facility characteristics:** Japanese staff and administration, Japanese food at least once a day, activities focused on Japanese culture, Japanese television as well as Japanese environment; home-like environment.<br>▪ **Non-Japanese oriented facilities (residents all JAs):** preference for some of the characteristics, e.g. Asian flavored food, rice some Japanese dishes<br>▪ **Food:** preference for Asian food in both RCFEs. Preference of Japanese food over American food.<br>▪ **House keeping services:** cleanliness in the facility.<br>▪ **Activities:** no preference for Japanese television. Preference for “own work” (to not feel useless).

Reference	Country & study design	Study population & Setting	Self or proxy reported	Preference category
				▪ **Care service models:** preference for "Japanese care" (eldercare as a calling) and showing respect.
▪ Hurley C., et al. 2013	▪ Australia<br>▪ Qualitative design - bilingual face-to-face (N=22) and four focus-group interviews (N=48) (phase 2) and telephone-interviews with community-based service provider (phase 1) (N=22)	▪ Greek elders (N=22)<br>▪ Age: 79 (mean)<br>▪ Sex: female N=15, male N=7<br>▪ Setting: Community-based service provider	▪ Self- and proxy-reported	▪ **House keeping service:** preference for higher standard (e.g. cleaning, frequent service) preference for Greek-speaking service providers<br>▪ **Informal care:** receiving informal assistance from family (family or informal care). When receiving formal care, preference to have family around to support.<br>▪ **Language:** older Greeks prefer to receive care from Greek-specific service (less language barriers)
▪ Ibrahim S. A., et al. 2004	▪ United States<br>▪ Qualitative design – focus group interviews with open-end questions (N=10)	▪ African Americans N=75<br>▪ Age: 62 (mean)<br>▪ Sex: female N=54 (72%)<br>▪ Setting: chronic knee or hip pain	▪ Self-reported	▪ **Religion:** preference for use of prayer or faith as pain management/care (only god can heal)<br>▪ **Ethnicity:** No clear preference; 10% preferred physician of the same race<br>▪ **Biological sex:** 18,2% participants noted a preference for the same sex
▪ Iwasaki M., et al. 2016	▪ United States<br>▪ Quantitative design - survey	▪ Japanese Americans (JA) N=264 and non-Japanese Americans (non-JA) N=232; total N=499<br>▪ Age: JA 60.20 (mean) and non-JA 59.45 (mean)<br>▪ Sex: female JA=67.42% and non-JA=70.21%<br>▪ Setting: long-term care (LTC) planning	▪ Self-reported	▪ **Informal care:** No preference for family care giving.<br>▪ **Care service models:** preference of non-cultural elements (e.g. transportation services, Internet access) over Japanese cultural-specific elements (e.g. Japanese cultural activities).<br>▪ **Ethnic Identity within the Community:** preference to be in a residential and LTC community where a mixture of Japanese and non-Japanese individuals reside.
▪ Johnson R.A., et al. 2001	▪ United States<br>▪ A Systematic Literature review	▪ African America, Caucasian and Latino elders<br>▪ Age: no information<br>▪ Sex: no information	▪ Review	▪ **Individual Living Arrangements:** nursing home as an acceptable alternative for care when family care was not possible.<br>▪ **Organizing Informal Care:** need of assistance by relying predominantly on their families [adult children], or on other informal sources, such as prayer, denial, displacement or worry.
▪ Lee E-H. 2012	▪ United States<br>▪ Qualitative design – three case studies, included interviews –	▪ Korean American elders N=26<br>▪ Age: average=78 (58 to 90)<br>▪ Sex: female N=21; male N=5	▪ Self- and proxy-reported	▪ **Facility Characteristics:** preference for a house or bedroom facing south.

Reference	Country &<br>study design	Study population &<br>Setting	Self or proxy reported	Preference category
	most in Korean - (open ended questions) with 26 Korean American elders, 6 relatives, and 5 staff members and observation	▪ Setting: Korean American nursing home environments and the needs, preferences, and levels of satisfaction of Korean American residents in ethnic nursing homes		▪ **language:** preferred staff who shared the same culture and language.<br>▪ **Activities:** Korean cable TV channels, Korean foods for their meals, and church services conducted by Korean pastors. Eat-time, sing-along, karaoke with Korean words on a large television screen, bingo, table games, birthday parties, whatu (a Korean flower card game), movies, crafts, Bible study, and dance and music. The residents were more likely to **<u>prefer</u>** informal (rather than formal) activities in the facility, including watching Korean TV programs and Korean movies, reading a Korean newspaper (this applied, in particular, to male residents), talking with other residents, playing hwatu (a Korean card game), and growing plants. the residents were much less likely to participate in the activity programs that were conducted in English. “well-planned activities relevant to residents‘ culture and preferences facilitate their involvement in the programs.”<br>▪ **Food:** Korean food<br>▪ **Herbal Remedies:** Asian herbal medicines would improve the tone of body organs and increase longevity<br>▪ **Facility characteristics:** underfloor heating in Korean housing; ondol room to a carpeted floor<br>▪ **Language:** staff from the same cultural and linguistic background to increase the quality of care and life.
▪ Lillekroken D., et al. 2024	▪ Norway<br>▪ A Systematic Literature Review<br>▪ Interviews, observations and surveys	▪ Older immigrants N=488<br>- Iranians<br>- Ghanaians<br>- Vietnamese<br>- Syrians<br>- Hindustani<br>- Chinese<br>- Africans<br>- Indians<br>- Pakistani	▪ Self-reported	▪ **Food:** preference for cultural food<br>▪ **Food and identity:** traditional food is part of the identity. For example, across all cultures, women tend to hold significant food-related roles associated with providing food for their families as wives and mothers.<br>▪ **Adapting to the host country´s food culture:** The longer they live in the host country, the easier it is to adopt food products specific to the host country

Reference	Country & study design	Study population & Setting	Self or proxy reported	Preference category
		- Bangladeshi<br>- Caribbean<br>▪ Age: 65 and older<br>▪ Sex: female: N=290; male N=198<br>▪ Setting: food habits and meal preferences of older immigrants		Younger immigrants change their food consumption after migration<br>No access to traditional food in nearby food shops<br>Changes in food habits because of a new environment (i.e., moving to a nursing home – institutional practices)
▪ Lim K., et al. 2024	▪ United States<br>▪ Mixed-methods research design: virtual semi-structured qualitative interviews (N=40) and survey (N=100)	▪ Care givers of older Asian Americans (qualitative study) N=40 and (quantitative study) N=100<br>Korean N=13<br>Asian Indian N=22<br>Chinese and Hong Kong N=46<br>Filipino N=9<br>Pakistani N=4<br>Vietnamese and Hmong N=20<br>Bangladesh N=1<br>Japanese N=17<br>Taiwanese N=8<br>▪ Age: care giver: age 20 or older and supported an Asian immigrant relative age 60 or older who had received any institutional or primary healthcare in the United States for at least one year before participation<br>▪ Sex: female N=107; male N=30<br>▪ Setting: traditional dietary preferences and the food offered in hospitals and long-term care environments	▪ Proxy-reported	▪ **Food:** preference for traditional Asian food
▪ Liu P., et al. 2021	▪ United States<br>▪ Mixed Methods research design—case study (phase 1) and a visual preference survey using 24 photographs (phase 2)	▪ Chines elderly immigrants (first generation) N=95<br>▪ Age: 82 (average)<br>▪ Sex: female N=61 (64%)<br>▪ Setting: outdoor walking in care facility	▪ Self-reported	▪ **Cultural familiarity:** Chinese classical style outdoor walking spaces (Chinese pathway)
▪ MacEntee M.I., et al. 2014	▪ Canada<br>▪ Qualitative Design – focus group interviews N=4	▪ Punjabi-speaking South-Asian immigrants N=33<br>▪ Age: between 65 and 75	▪ Self-reported	▪ **Herbal Remedies:** preference for Indian substances, use only having slight problems with teeth.

Reference	Country & study design	Study population & Setting	Self or proxy reported	Preference category
		▪ Sex: female N=17, male N=8, unknown N=8<br>▪ Setting: oral health		Preference for a mix of traditional remedies supplemented, if possible, by elective oral health care in India, and by emergency dental care in Canada.<br>▪ **Medicine:** preference strong for western medicine when having pain.<br>▪ **Language:** preference for a dentist from Punjabi community, easier way to explain the problem (often language barriers).
▪ Matsuoka A.K. 1999	▪ Canada<br>▪ Multi-methods design (qualitative and quantitative) – formal interviews, semi-structured taped interviews	▪ Japanese Canadians total N=41 Issei N=20 (immigration before 1950) Nisei N=21 (2nd generation/children)<br>▪ Age: 62 to 96<br>▪ Sex: Issei female N=10 and male N=10; Nesei female N=10 and male N=11<br>▪ Setting: care in later life	▪ Self-reported	▪ **Individual living arrangements:** children (daughters) or family as source of care. Issei women express interest in using services in combination with preference for caregiving children<br>▪ **Gender:** female member of the family who is selected to care for older people.<br>▪ **Ethnic Identity within the Community:** preference for Japanese nursing homes.
▪ Minde G.T. 2021	▪ Norway<br>▪ Qualitative design - Case studies (Ethnographic interviews and participant observation)	▪ Older Sámi<br>▪ Age: female: 86; male: 85<br>▪ Sex: female N=1; male N=1<br>▪ Setting: Experiencing memory impairment in late life	▪ Self-reported	▪ **Activities:** Sámi residents prefer to have activities which remains them about elements from their Sámi way of living.
▪ Min J.W. 2005	▪ United States<br>▪ Quantitative design - a cross-section survey (in Korean language as well)	▪ Korean Americans N=144 (immigrated after 1965)<br>▪ Age: 75.5 (mean)<br>▪ Sex: female N=86; male N=58<br>▪ Setting: hip fracture and stroke / Long-term Care Arrangements	▪ Self-reported	▪ **Individual living arrangements:**<br>1) preference for mixed arrangements (e.g. home health care, receiving care from professionals at children's home)<br>2) preference for formal care arrangement (caregiver and care location) N=51%; preference for informal care arrangement N=28% and mixed-arrangement N=21%<br>3) stronger preferences for "all informal" or "mixed" care arrangement for short-term care
▪ Min J.W., et al. 2009	▪ United States<br>▪ Quantitative design - cross-sectional survey	▪ Mexican American (Latinos) N=89 and non-Latino Whites N=30; total N=119	▪ Self-reported	▪ **Individual living arrangements:** Mexican-American elders (55%) preferred to rely on a formal/professional helper, while 45% would turn to informal caregivers or helper when faced

Reference	Country & study design	Study population & Setting	Self or proxy reported	Preference category
		▪ Age (mean): MA 73.1 and non-Latino Whites 72.8<br>▪ Sex: female MA=79.8% and non-LW=80.0%<br>▪ Setting: hip fracture and use of LTC services		with care needs following hip fracture. non-Latino White elders mainly preferred (83.3%) formal/professional helper.<br>▪ **Organizing informal care:** family caregiving and that children should be responsible for elders care (Mexican American)
▪ Montayre J., et al. 2019	▪ New Zealand<br>▪ Qualitative design – face-to-face interviews	▪ Filipino immigrants N=15<br>▪ Age: 60-64 N=4; 65 and older N=11<br>▪ Sex: female N=8, male N=7<br>▪ Setting: living and care arrangements	▪ self-reported	▪ **Food:** Filipino food<br>▪ **Care Service Models**: to maintain traditional practices and upholding Filipino spiritual and cultural beliefs<br>▪ **Individual living arrangements:**<br>1) a residential aged care admission when no longer able to live independent<br>2) to live with the family, realizing that they might be not able to take care<br>3) going back to Philippines (when its not possible to live with the family)
▪ Ness T. M., et al. 2020	▪ Sweden<br>▪ Qualitative design - semi-structured interview	▪ South Sami (indigenous population) N=56<br>▪ Age: 74 (median)<br>▪ Sex: female N=31, male N=25<br>▪ Setting: Nursing home care (perspective on receiving care in the future)	▪ Self-reported	▪ **Same language:** care provider who should speak South Sami while providing care in a nursing home [better understanding of language and culture]<br>Preference for Sami caregiver "because of the risk that they might go back to their first language if suffering from dementia".<br><u>no preference</u>, when participants do not speak Sami themselves or are bilingual (they speak Swedish) [disowning to have a South Sami background/adopted the Swedish culture].<br>▪ **Food:** Sami food and the wish for assistance in preparing Sami food when elders no longer could do this for themselves
▪ Netto G. 1998	▪ United Kingdom/Scotland<br>▪ Qualitative design - interviews	▪ Minority Ethnic carers of older people N=45<br>Afro-Caribbean N=2<br>Bangladeshi N=3<br>Chinese N=15<br>Indian N=8<br>Pakistani N=15	▪ Self- and proxy-reported	▪ **Same language:** sitter to speak the same language.<br>▪ **Gender:** preference for the same gender as those they [carers] were looking after.<br>▪ **Ethnic identity within the community:** preference for being together with other people

Reference	Country & study design	Study population & Setting	Self or proxy reported	Preference category
		Other Asian N=2<br>▪ Age: above 45<br>▪ Sex: female=75%<br>▪ Setting: respite services; perspective of the carers who care for elder family members		form ethnic minorities speaking the same language.<br>▪ **Food:** culturally familiar food [meet any religious and dietary requirements].
▪ O'Dwyer M., et al. 2024	▪ Australia<br>▪ Quantitative design - secondary analysis of the Australian Institute of Health and Welfare (AIHW)	▪ Permanent aged care facility residents born in a non-main English-speaking country N= 47,796<br>▪ Age: 70 years and older<br>▪ Sex: no information<br>▪ Setting: residential aged care facilities	▪ Not reported	▪ **Language:** people living in residential aged care were born in a non-main English-speaking country and have a preference for a language other than English.<br>➔ While same-language support exists, linguistic minorities within care facilities remain vulnerable to isolation due to cultural and social differences, even among co-language speakers. Cultural diversity within a language group can further exacerbate this isolation.
▪ Pasco A. C., et al. 2004	▪ Canada<br>▪ Qualitative design – ethnographic interviews (in English or Filipino)	▪ Filipino Canadians N=23<br>▪ Age: 33 to 86<br>▪ Sex: female N=13, male N=11<br>▪ Setting: care in Canadian hospitals; nurse-patient relationship	▪ Self-reported	▪ **Specific perspectives on ethnicity:** caring by a nurse from the own culture. nurse to be *hindi ibang tao* (to become "*one of us*", nurses have to share common identity and experiences, e.g. being an immigrant or similar family roles OR spending time with patient and using the languages of word, touch, gaze, and food) to disclose needs.<br>▪ **Organizing Informal Care:** family to assist/care**.**<br>▪ **Same language:** to be cared by a nurse from the same culture, because language can also include facial expressions, gestures, speech intonation, volume, and colloquialisms, and these can be misinterpreted in across-cultural context, leads to loss of free expression.<br>▪ **Leisure activities:** Food-sharing is a traditional way of socializing in the Philippines. Preference for giving food primarily as a means of reciprocating for care.<br>▪ **Food:** preference for favorite food and Filipino dishes

Reference	Country & study design	Study population & Setting	Self or proxy reported	Preference category
▪ Pasquali E. A. 1985	▪ United States<br>▪ Qualitative design - anthropological fieldwork	▪ Elderly Cuban immigrants (refugees) N=355<br>▪ Age: No information<br>▪ Sex: No information<br>▪ Setting: eating habits	▪ Self-reported	▪ **Food** (helps to self-identify as Cuban)**:** typical Cuban Food [e.g. white rice and black beans, pork, pig roast). *Café con leche* preferred in the morning and *espresso* after lunch.
▪ Polacsek M. 2016	▪ Australia<br>▪ Qualitative design - semi-structured interviews (face-to-face and one telephone interview)	▪ German Australians N=15 (migration between 1945 and 1965)<br>▪ Age: 65 to 90<br>▪ Sex: female N=8, male N=7<br>▪ Setting: support needs	▪ Self-reported	▪ **Organizing informal care:** No preference for family caregiving.<br>▪ **Language:** No preference for German language<br>▪ **Facility characteristics:** No preference for German residential service.<br>▪ **Leisure activities:** Preference for German traditions e.g., German delicatessen and celebrating Christmas Eve.
▪ Rhodes P., et al. 2003	▪ United Kingdom<br>▪ Qualitative design - in-depth interviews (11 in Sylheti and one in English)	▪ Bangladeshi immigrants N=12<br>▪ Age: 68 (mean)<br>▪ Sex: female N=8, male N=4<br>▪ Setting: Diabetes care	▪ Self-reported	▪ **Organizing informal care:** to rely on relatives, even when alternatives were available e.g., as interpreter.<br>▪ **Language:** to take children/other family members to appointments because of strong language barriers (strong dependency/reliance on children).<br>▪ **Same language:** No clear preference for a Bengali-speaking doctor. Female patients N=2 preferred the approach of the English nurse and not the Bengali-speaking doctor (because she was a woman, but also because she was more approachable and prepared to try to listen to their concerns)
▪ Roberts L. R., et al. 2015	▪ United States<br>▪ Qualitative design – Semi-structured key informant interviews and focus groups (FG) N=75<br>1) 15 interviews with health care professionals and community-based agencies serving older adults<br>2) 8 FGs included family caregivers and physician	▪ African American N=16, Latinos N=22 and Whites N=19 Family-caregiver N=8 and physicians N=10 Total N=75<br>▪ Age: 55 to 78<br>▪ Sex: not known<br>▪ Setting: Perceptions of Aging and Cognitive Decline	▪ Self- and proxy-reported	▪ **Organizing informal care:** Preference for family caregiver (spouse or a child). African American and especially Latinos express community-oriented, collectivistic view of caring for elders.<br>▪ **Care services models:** report that Asians and Latinos prefer a home care model and Whites prefer an "out-of-home"-care model.<br>▪ **Care services models:** Preference for family member to join to appointments<br>▪ **Gender:** African American prefer female family caregivers

Reference	Country & study design	Study population & Setting	Self or proxy reported	Preference category
	3) 6 FGs with Latino, African American, and White older adults			
▪ Runci S. J., et al. 2005	▪ Australia<br>▪ Quantitative design - postal questionnaire (sent to 189 registered aged care facilities)	▪ Older persons from culturally diverse backgrounds<br>▪ Age: not known<br>▪ Sex: not known<br>▪ Setting: Language needs and service provision for culturally diverse older persons in residential care facilities	▪ Proxy-reported (directors responded the questionnaires)	▪ **Same laguage:** Preference for a non-English language<br>▪ **Care service models:** preference for culturally specific services (language specific, Activity programs, food)<br>▪ **Language** (most common): Dutch(N=224), Greek (N=207), Polish (N=144), Russian (N=142),Italian (N=97), Yiddish (N=79) and German (N=43)
▪ Sciegaj M., et al. 2006	▪ United States<br>▪ Qualitative design – face-to-face interviews (30-40 min)	▪ African American (AA) N=200; Chines American N=200; Latino N=131, White Western-European American N=200<br>▪ Age: AA mean age=80; Latinos mean age=75; Chinese Americans mean age=77; WWEA mean age=77<br>▪ Sex: female AA=74%; female Latinos=69%; female Chines Americans=73%; female WWEA=71%; total female=73%<br>▪ Setting: receiving services from the Massachusetts Home Care Program or home health aide services	▪ Self-reported	▪ **Care service models:** 1) Latino elders expressed the strongest desire for control over their services. 2) AA expressed the strongest desire about their workers and for making decisions regarding their services without assistance from family of professional agency staff.<br>▪ **Care service models:** Preference for the different new approach models<br>▪ **Care service models:** Preference for Complete Control Over Service Areas (CLTC decisions, services and worker-related issues)
▪ Shin D.S. 2008	▪ United states<br>▪ Qualitative design – interviews with open end questions	▪ older Korean Americans N=12<br>▪ Age: 65 and older<br>▪ Sex: female N=8, male N=4<br>▪ Setting: residential and caregiver preferences (recruited from Korean church N=3, nursing home N=5 (diagnosed with dementia) and senior housing N=4)	▪ Self-reported	▪ **Organizing informal care:** female family member (daughter or daughter in law)<br>▪ **Individual living arrangements:** living independently in senior housing or co-residing with their adult children to living in a nursing (if becoming bedridden).<br>▪ **Cultural familiarity:** the availability of culturally tailored services was one of the key factors of not preferring co-residents with children.<br>▪ **Individual living arrangements:** older Korean may not want to live in a nursing home but

Reference	Country & study design	Study population & Setting	Self or proxy reported	Preference category
				changing circumstances might make it unavoidable<br>▪ **Specific perspectives on ethnicity:** staff members of the same culture/ethnicity and share a cultural background.<br>▪ **Principles of house-keeping services:** Preference for more cleanliness.
▪ Shrestha S., et al. 2023	▪ Norway<br>▪ A literature review - a meta-ethnography study	▪ Caregiver of older immigrant family members with and without care needs<br>▪ Age: no information<br>▪ Sex: no information<br>▪ Setting: experiences of individuals caring for older family members with immigrant backgrounds	▪ Self- and proxy-reported	▪ **Individual living arrangements:** Older family members preferred co-residence with their family members<br>▪ **Informal care:** Many of the older family members preferred to be cared for by their children than others. Strong care norms i.e., the perceived responsibility of caring for family members (moral obligation of caregiving)<br><br>**moral dilemma:** *A few caregiver participants, who migrated to Europe with their parents in early childhood, asserted that they will be less dependent on their children in their old age, and said the younger generation (who were born and raised in Europe) may not be so dedicated to the caring norms espoused by older family members.*<br>▪ **Care service models:** preference for their older relatives to die at home surrounded by relatives because of fear that outsiders would not provide the same level of care as family members<br>▪ **Care service models:** preference for cultural sensitivity within health services.
▪ Shrestha S., et al. 2024	▪ Norway<br>▪ Qualitative design - semi-structured interviews N=18 and two group interviews in Urdu and English	▪ Caregiver of older Pakistani migrants N=19<br>▪ Age: 25-62<br>▪ Sex: female N=19<br>▪ Setting: formal and informal care and preferences regarding the place of care for older family members with migrant backgrounds	▪ Proxy-reported	▪ **Organizing informal care:** Preferences for home with the possibility of sharing care<br>▪ **Individual living arrangements:** Perceived preferences of seniors for staying at home and live together with the adult children<br>▪ **Gender:** Older parents' preferences were for same-sex nurses for personal hygiene assistance

Reference	Country & study design	Study population & Setting	Self or proxy reported	Preference category
				"We had requested that no male nurse should be sent for her because my mother-in-law did not like that a male nurse would touch her. When they [Home service nurse from municipality] would call me to send male nurse, then I used to say that she will not even give him to enter her room, so instead I used to do her cleaning, changing diapers, and bathing."<br><br>▪ **Food:** preference for traditional food<br>▪ **Activities**: daily activities, Pakistani TV programs, prayer, and gender-matching services for older adults<br>▪ **Individual living arrangements:** Preferences for nursing homes emerged when care was reduced to physical assistance, particularly when older adults were bedridden and unable to move. Caregivers preferred future nursing home care due to their adaptation to Norwegian life and reduced language barriers.
▪ Sudha S. 2014	▪ United States<br>▪ Qualitative design – interviews	▪ Asian Indians (families, with at least one senior elderly N=5 than 60 and midlife adult children N=19) total N=24<br>▪ Age: Elderly above age 60<br>▪ Sex: Elderly female N=3, male N=2; Midlife adults female N=9, male N=10<br>▪ Setting: Care arrangements/ views and expectations of elder care among Asian Indian seniors and their midlife children,	▪ Self- and proxy-reported	▪ **Organizing informal care:** preference for children as caregiver (elderly)/ rely on family Midlife adults have no preference anymore their families/children to care for them.<br>▪ **Individual living arrangements:** No preference for institutional care instead preference to go back to India.<br>▪ **Individual living arrangements:** preference for living near the children not with them<br>▪ **Ethnic identity:** Most of the older participants preferred to join community specific cultural groups<br>▪ **Food:** culturally food
▪ Sudha S., et al. 1999	▪ United States<br>▪ Quantitative design - survey questionnaires	African Americans N=283 and White Americans N=254; Total N=537<br>▪ caregiver of elderly (sample) N=361 and caregiver of those who were not able to participate N=146 (15 caregiver dropped	▪ Self- and proxy-reported	▪ **Organizing informal care:** African Americans are more likely than Whites to express a preference for family care. Older people and men prefer family care more. Higher socio-economic status is associated with less preference for family care.

Reference	Country & study design	Study population & Setting	Self or proxy reported	Preference category
		because of other ethnicity) AA N=265 and WA N=227<br>▪ Age: 75 (mean)<br>▪ Sex: female=71%, male=29%<br>▪ Setting: attitudes toward rest home placement		
▪ Suurmond J., et al. 2016	▪ Netherlands<br>▪ Qualitative design - semi-structured group interviews N=50 and individual interviews N=5 (in preferred language) [Moroccan and Turkish group, women and men were interviewed in separate groups]	▪ Turkish N=21, Moroccan N=11, Surinamese N=14 and ethnic Dutch N=8<br>▪ Age: 52-81<br>▪ Sex: Turkish female N=10, male N=11; Moroccan female N=4, male N=8<br>▪ Setting: use of home care services	▪ Self-reported	▪ **Organizing informal care:** Preference for family to provide care/ take care of their needs<br>▪ **Organizing informal care:** Ethnic- Dutch participants- expressed a preference for women to take care of needs of the elderly family members.<br>▪ **Individual living arrangements:** ethnic minorities only considered home care service when the family members hardly coped anymore.<br>▪ **Same language:** Ethnic minorities preferred a home care provider who spoke their language
▪ Wu S., et al. 2008	▪ United States<br>▪ Qualitative design – 20 meal observations and interviews with 7 residents, 9 family members and 17 staff members	▪ Chinese Americans N=7 (plus 9 family members and 17 staff members (Chinese N=11; Filipino N=2, Other N=4))<br>▪ Age: mean 81<br>▪ Sex: 1) residents female N=5, male N=2; 2) staff members female N=12, male N=5;<br>▪ Setting: Nursing home	▪ Self- and proxy-reported	▪ **Food preference:** provision of culturally competent mealtime care for ethnic Chinese long-term care residents involves important food service practices in addition to kinds of food.
▪ Xiao L.D., et al. 2022	▪ Australia<br>▪ A literature review - A meta-synthesis of qualitative research	▪ ethnic minority residents'; nursing home staff and family caregivers<br>▪ Age: no information<br>▪ Sex: no information<br>▪ Setting: ability to exercise their autonomy in communication and care while in nursing homes	▪ Self- and proxy-reported	▪ **Language:** to express their preferences in their first language<br>▪ **Specific perspectives on ethnicity:** required staff to demonstrate cultural desire to overcome difficulties in meeting residents' care needs.<br>▪ **Same language:** Finnish older people wanted to stay at a nursing home completely with well-trained Finnish-speaking staff because of the risk that older people remember only their mother tongue in association with dementia. Native language instruction facilitated smoother nursing care and daily life for non-Swedish speaking residents.

Reference	Country & study design	Study population & Setting	Self or proxy reported	Preference category
				▪ **Leisure activities:** Finnish parish which held church services, and by the Sweden–Finnish Association which booked choir concerts. Singing was a common activity among the residents. Preservation of familiar Finnish customs and habits.<br>▪ **Food:** preference for cultural traditional food and food habits
▪ Xiao L.D., et al. 2023	▪ Australia<br>▪ Qualitative design - individual and focus group interviews	▪ Residents N=24; family members N=5:<br>Australian N=9<br>Greek N=5<br>German N=1<br>Italian N=8<br>English N=6<br>▪ Age: mean=86,3<br>▪ Sex: female N=21; male N=8<br>▪ Setting: factors affecting residents fulfilling self-determination in ethno-specific and mainstream nursing homes.	▪ Self-reported	▪ **Language:** use the language of choice to freely express their care needs and preferences.<br>▪ **Interpreter:** culturally competent bilingual staff to provide interpreter services was a basic condition for CALD residents to meet their care needs and preferences in ethno-specific nursing homes where a large proportion of staff did not share the same language and culture with them.<br>▪ **Same language:** preference for staff, therapist and health care professionals who speak their language<br>▪ **Leisure activities:** Those activities indicate the ethno-specific nursing homes' connections with community organizations by which they were able to provide resources needed to meet residents' expectations for maintaining their religious activities they had through their live course.
▪ Yeung E. H., et al. 2015	▪ Canada<br>▪ Qualitative design – Interviews (in Cantonese and English) N=18	▪ Chinese-Canadian N=5 and family-caregivers N=13<br>▪ Age: Elderly 68; Caregiver 60<br>▪ Sex: Elderly female N=3; Caregiver female N=6<br>▪ Setting: stroke survivors and re-integration back in the community / recovery and rehabilitation	▪ Self-reported and proxy-reported	▪ **Same language:** preference for staff, therapist and health care professionals who speak Chinese.<br>▪ **Food:** traditional Chinese food (Rice, Congee, Noodles) while being on a diet after stroke. Believe that 'yin' or 'yang' foods would help treat illness like stroke.<br>▪ **Medicine:** Preference for traditional Chinese medicine.
