# Supplementary material for: Care preferences of older migrants and minority ethnic groups with various care needs: A scoping review
Source: PLoS One. 2026 Jan 23;21(1):e0341147. doi: 10.1371/journal.pone.0341147 (PMC12829939; doi:10.1371/journal.pone.0341147)
Supplement: S2 Table — (PDF) [file pone.0341147.s002.pdf]

Supplementary table 2: Search strategy in MEDLINE (via PubMed)

	Search Terms	
**Population**	#1 **aged[MeSH Terms])**<br>#2 **aged[Title/Abstract]**<br>#3 **retired[Title/Abstract]**<br>#4 **older[Title/Abstract]**<br>#5 **resident*[Title/Abstract]**<br>#6 **elder*[Title/Abstract]**<br>#7 **senior*[Title/Abstract]**<br>#8 **transients and migrants[MeSH Terms]**<br>**#9 immigra*[Title/Abstract]**<br>**#3 ethnic*[Title/Abstract]**<br>**#4 culture[MeSH Terms]**<br>**#5 transnational[Title/Abstract]**<br>**#6 emigra*[Title/Abstract]**<br>**#7 cultural diversity[MeSH Terms]**<br>**#8 migration background"[Title/Abstract]**<br>#9 **migrant*[Title/Abstract]**<br>**#10 immigrant[Title/Abstract]**	
**Concept**	**#11 patient preference[MeSH Terms]**<br>**#12 "preference-based"**<br>**#13 preferences**<br>**#14 prefer***	
**Context**	**#15 care[Title/Abstract]**<br>**#16 patient care[MeSH Terms]**<br>**#17 nursing[Title/Abstract]**	
**Search String**	(((((((aged[MeSH Terms]) OR (aged[Title/Abstract])) OR (retired[Title/Abstract])) OR (older[Title/Abstract])) OR (resident*[Title/Abstract])) OR (elder*[Title/Abstract])) OR (senior*[Title/Abstract])) AND ((((((((((((transients and migrants[MeSH Terms]) OR (immigra*[Title/Abstract])) OR (ethnic*[Title/Abstract])) OR (culture[MeSH Terms])) OR (transnational[Title/Abstract])) OR (emigra*[Title/Abstract])) OR (cultural diversity[MeSH Terms])) OR ("migration background"[Title/Abstract])) OR (migrant*[Title/Abstract])) OR (immigrant[Title/Abstract])) AND ((((patient preference[MeSH Terms]) OR ("preference-based")) OR (preferences)) OR (prefer*))) AND (((care[Title/Abstract]) OR (nursing[Title/Abstract])) OR (patient care[MeSH Terms])))	
