# Supplementary material for: Care preferences of older migrants and minority ethnic groups with various care needs: A scoping review
Source: PLoS One. 2026 Jan 23;21(1):e0341147. doi: 10.1371/journal.pone.0341147 (PMC12829939; doi:10.1371/journal.pone.0341147)
Supplement: S3 Table — (PDF) [file pone.0341147.s003.pdf]

Supplementary table 3: Search strategy in PsychInfo (via EBSCO)

	Search Terms	
**Population**	**S1 MH aged**<br>**S2 TI aged OR AB aged**<br>**S3 TI retired OR AB retired**<br>**S4 TI older OR AB older**<br>**S5 TI resident* OR AB resident***<br>**S6 TI elder* OR AB elder***<br>**S7 TI senior* OR AB senior***<br><br>**S9 TI immigra* OR AB immigra***<br>**S10 TI ethnic* OR AB ethnic***<br>**S11 MH culture**<br>**S12 TI transnational OR AB transnational**<br>**S13 TI emigra* OR AB emigra***<br>**S14 MH cultural diversity**<br>**S15 TI migration background OR AB migration background**<br>**S16 TI migrant* OR AB migrant***	
**Concept**	**S19 MH patient preference**<br>**S20 TI preference-based OR AB preference-based**<br>**S21 TI prefer* OR AB prefer***	
**Context**	**S24 TI care OR AB care**<br>**S25 TI nursing OR AB nursing**<br>**S26 MH patient care**	
**Search String**	**S8:** S1 OR S2 OR S3 OR S4 OR S5 OR S6 OR S7<br><br>**S17:** S9 OR S10 OR S11 OR S12 OR S13 OR S14 OR S15 OR S16<br><br>**S18:** S8 AND S17<br><br>**S22:** S19 OR S20 OR S21<br><br>**S23:** S18 AND S22<br><br>**S27:** S24 OR S25 OR S26<br><br>**S28:** S23 AND S27	
