# Supplementary material for: Care preferences of older migrants and minority ethnic groups with various care needs: A scoping review
Source: PLoS One. 2026 Jan 23;21(1):e0341147. doi: 10.1371/journal.pone.0341147 (PMC12829939; doi:10.1371/journal.pone.0341147)
Supplement: S6 Table — (PDF) [file pone.0341147.s006.pdf]

Supplementary Table 3: Summary of terms used in the included studies

Population term	Study populations	Notes on conceptual meaning	Authors
**Immigrants**	Arab-Americans; Chinese immigrant elders; Pakistani immigrants; Punjabi-speaking South-Asian; Filipino immigrants; Africans; Indians; Bangladeshi; Caribbeans; Cubans; Africans, Asians and South Americans living in Europe; Asian Indians; Latinos; non-Latino Black;	Individuals who have moved across international borders and settled long-term or permanently in the host country, often with legal residency or citizenship. The term emphasizes settlement and adaption to the host society. Immigrants may also belong to minority ethnic groups depending on social inclusion and cultural affiliation.	(Ajrouch, 2005); (Arora et al., 2020); (Liu et al., 2021); (MacEntee et al., 2014); (Montayre et al., 2019); (Lillekroken et al., 2024); (Pasquali, 1985); (Shrestha et al., 2023);(Sudha, 2014); (Aroian et al., 2005); (Alley et al., 2016); (Lim et al., 2024)
**Migrants**	Older migrant communities: Pakistani, Italian, Bangladeshi, Polish, Indian, Ukrainian, Hungarian, African Caribbean; Greek elders; older Pakistani; German Australians;	A broad umbrella term referring to individuals who have crossed national boarders, regardless of the reason, duration, or legal status. The term highlights processes of mobility, transitions, and ongoing cultural negotiation rather than formal citizenship.	(Giuntoli & Cattan, 2012); (Hurley et al., 2013); (Shrestha et al., 2023); (Polacsek & Angus, 2016)
**Ethnic Minority/ Ethnic Minorities**	African Americans; Norwegians, Black South African; Japanese American women; Korean American; Latinos; Moroccans; Turkish; Surinamese;	Groups that differ culturally, linguistically, or religiously from the dominant population, whether or not they have a migration background. The concept reflects issues of social inequality, discrimination, and the need of culturally appropriate healthcare.	(Dupree et al., 2005); (Hanssen & Kuven, 2016); (Lee, 2010); (Roberts et al., 2015); (Suurmond et al., 2016);
**Minority Ethnic Group(s)**	Japanese Canadians; Korean elders; Japanese American; African American; Chinese American; Latinos;	Used to describe numerically smaller ethnic populations within the national population. The term focuses on diversity, inclusion, and cultural recognition rather than on migration history and may include both migrants and non-migrant groups.	(Matsuoka, 1999); (Min, 2005); (Iwasaki et al., 2016); (Sciegaj, 2006)
**Ethnic Elders/ Minority Elders**	Ethnic Chinese migrants; Black or African Americans, Hispanics, Latino elders; non-Latino Black; Mexican-American; Elderly African Americans;	Older adults belonging to ethnic minority backgrounds. The term addresses the intersection of aging, cultural identity, language, and care needs.	(Chan & Quine, 1997); (Dickson et al., 2013); (Dole et al., 2000); (Hefele et al., 2016); (Min & Barrio, 2009); (Johnson & Tripp-Reimer, 2001); (Ibrahim et al., 2004); (Sudha & Mutran, 1999)
**Indigenous People/Communities**	Great Lake American Indians; Indigenous people living in Navaho reservation; older Sami; South Sami;	Populations with historical continuity to pre-colonial societies and distinct cultural, linguistic, and territorial identities. They are not migrants but often experience structural inequalities and require culturally safe, community-based care approaches.	(Chapleski et al., 2003); (Battistone et al., 1998); (Minde, 2021); (Ness et al., 2020)
**Culturally and Linguistically diverse (CALD) Populations**	People with CALD: Asians, Europeans, Indians, Iranian, Mexican-American, Indigenous people; non-main English-speaking; Persons from CALD background;	An Australian policy and research term describing people whose first language and/or cultural background differs from that of the majority population. It emphasizes language, communication, and cultural competence in care and inclusion.	(Gaviola et al., 2024); (O'Dwyer et al., 2024); (Runci et al., 2005); (Xiao et al., 2022)
**No term**	People of Bangladeshi origin; Chinese elders; Participants from Greek/Italian/main-stream ethno-specific nursing home; Chinese Canadian; Filipino Canadian;	Studies that identified participants only by ethnicity or nationality, without applying a conceptual label (e.g., “migrant” or “minority”). This reflects the ongoing inconsistency and context-dependence of terminology in intercultural care research.	(Rhodes & Nocon, 2003); (Wu & Barker, 2008); (Chappell & Lai, 1998); (Xiao et al., 2023);(Yeung et al., 2015); (Pasco et al., 2004)
